# Neural synchrony within the motor system: what have we learned so far?

**DOI:** 10.3389/fnhum.2012.00252

**Published:** 2012-09-04

**Authors:** Bernadette C. M. van Wijk, Peter J. Beek, Andreas Daffertshofer

**Affiliations:** MOVE Research Institute, Faculty of Human Movement Sciences, VU University AmsterdamAmsterdam, Netherlands

**Keywords:** neural synchronization, oscillations, information processing, motor control, movement, motor cortex, corticospinal coherence, movement disorders

## Abstract

Synchronization of neural activity is considered essential for information processing in the nervous system. Both local and inter-regional synchronization are omnipresent in different frequency regimes and relate to a variety of behavioral and cognitive functions. Over the years, many studies have sought to elucidate the question how alpha/mu, beta, and gamma synchronization contribute to motor control. Here, we review these studies with the purpose to delineate what they have added to our understanding of the neural control of movement. We highlight important findings regarding oscillations in primary motor cortex, synchronization between cortex and spinal cord, synchronization between cortical regions, as well as abnormal synchronization patterns in a selection of motor dysfunctions. The interpretation of synchronization patterns benefits from combining results of invasive and non-invasive recordings, different data analysis tools, and modeling work. Importantly, although synchronization is deemed to play a vital role, it is not the only mechanism for neural communication. Spike timing and rate coding act together during motor control and should therefore both be accounted for when interpreting movement-related activity.

## Introduction

Oscillatory activity in the human cortex has been a focus of research ever since Hans Berger observed a strong alpha rhythm that modulates with opening and closing of the eyes or, more general, with visual attention (Berger, 1929). It soon was discovered that this rhythm originates from the occipital lobes (Adrian and Matthews, 1934a) and can be dissociated from another alpha rhythm close to the central sulcus, where also strong beta oscillations can be found that show suppression upon tactile stimulation (Jasper and Andrews, 1938). Later studies would reveal that these Rolandic rhythms also modulate with passive, voluntary and imagined movements (Jasper and Penfield, 1949; Gastaut, 1952; Chatrian et al., 1959). These observations prompted the by now traditional classification of frequency bands and the search for a relation with human functioning.

Oscillations picked up via EEG or MEG represent synchronous activity of many thousands of anatomically aligned neurons. Much work has been done to reveal the mechanisms underlying this synchronized, oscillatory firing (Adrian and Yamagiwa, 1935; Freeman, 1975; Hogan and Fitzpatrick, 1988; Lopes Da Silva, 1991; Basar, 1998). Cortico-thalamic loops (Lopes Da Silva et al., 1974; Steriade et al., 1990; Lumer et al., 1997a,b; Hughes and Crunelli, 2005), as well as the interplay between inhibitory interneurons and pyramidal cells (Lytton and Sejnowski, 1991; Cobb et al., 1995; Whittington et al., 1995; Buzsáki, 2006), proved to be crucial factors in this regard. In fact the mere anatomical architecture can yield self-sustaining rhythmic activity, i.e., neural oscillations in the absence of any (non-constant) input, which triggered the idea that alpha and beta oscillations act as an “idling” rhythm (Adrian and Matthews, 1934b; Pfurtscheller et al., 1996). It is currently believed, however, that the amplitude of the alpha rhythm signifies the degree of cortical inhibition (Klimesch et al., 2007), and there are indications for a similar role for the beta rhythm (Engel and Fries, 2010).

Oscillations may not only change locally due to an altered amplitude of (de-)synchronization within a population, but also display distinct patterns of synchronization with distant populations. It has been suggested that these long-range synchronization patterns contribute to the solution of the so-called “binding problem”: neural activity must be synchronized or “bound” so that their integration can represent a “gestalt” or pattern (Eckhorn et al., 1988; Gray et al., 1989; see also Singer and Gray, 1995 for an overview). For example, neural populations in visual cortex distinctively respond to features like color, shape, and motion. These features need to be integrated in order to form a compound percept, which, supported by various invasive recordings, is achieved through synchronization between neurons that encode different features of the same percept (Eckhorn et al., 1988; Engel et al., 1991; Kreiter and Singer, 1996; Fries et al., 1997). Precise timing of action potentials ensures maximal impact on a target population when its fluctuations in membrane potential reach a depolarized state. Input outside this time window is less effective. It may therefore yield a selection mechanism for competing oscillatory input (Roelfsema et al., 1996; Womelsdorf et al., 2007). For inter-areal distances longer than 2 mm, oscillations may have an important role in establishing in-phase synchronization (Konig et al., 1995).

While the relevance of synchronized oscillations for feature binding can be readily extended from visual processing to other sensory modalities like the olfactory (Freeman, 1978) or the auditory system (Aertsen et al., 1991; Vaadia et al., 1991), for the motor system this seems less obvious. One may view temporal binding as a more gestalt-like definition of motor programs, represented through cell assemblies (Wickens et al., 1994; Hommel, 2004). Alternatively, oscillations in the motor cortex may just modulate with attention to fine-tune sensorimotor control (Murthy and Fetz, 1992; Farmer, 1998). In either case, successful motor functioning depends on the interactions between multiple regions, including frontal, parietal, cerebellar, and subcortical areas, as well as the spinal cord. Proper timing of these interactions may well be critical. If synchronization is indeed an effective means for neural communication (Varela et al., 2001), then one can expect that also the motor system capitalizes on this capacity (Singer, 1994). In fact, alpha/mu, beta, and gamma oscillations do show strong movement-related modulations in large parts of the motor system (see, e.g., Ohara et al., 2001; Gross et al., 2005; Pollok et al., 2005; Cheyne et al., 2008; Houweling et al., 2008; van Wijk et al., 2012).

What do these modulations tell us about motor control? How do they lead to coordinated movements? How is information transferred to the spinal cord? The aim of this review is to answer these questions by offering a comprehensive but not necessarily complete overview of experimental results and to discuss what we believe are current key findings. Previous reviews focused primarily on more isolated aspects of synchronization in the motor system, for instance, the communication between sensorimotor cortex and spinal cord (Brown, 2000; Salenius and Hari, 2003; Baker, 2007), the role of beta oscillations (Engel and Fries, 2010; Jenkinson and Brown, 2011), physiological and pathological tremor (Schnitzler et al., 2006), and other disease states (Brown, 2007). By contrast, we here intend to cover the entire functional spectrum of synchronization in motor control. For this we integrate findings from multiple recording techniques, frequency bands, and (sub-)cortical regions of the motor system. We sketch these studies in light of current debates and methodological challenges regarding the interpretation of movement-related synchronization patterns.

## M1 oscillations

The threshold for evoking movements by electrical stimulation is lowest in the primary motor cortex (Brodmann's area 4 or simply M1) (Fritsch and Hitzig, 1870; Penfield and Boldrey, 1937). Mapping studies have revealed a somatotopical organization in M1 with projections to either a single motor unit pool or a group of muscles, and functionally related pyramidal tract neurons (PTNs) that appear to be organized in small clusters (Asanuma et al., 1979). The encoding of movement patterns occurs via intricate connections within and between cortical modules, of which PTNs only form a minority of cells (Keller, 1993). Here, we focus in particular on empirical evidence for a possible relation between oscillatory activity observed in M/EEG and the activity of PTNs (targeting the spinal cord). Can oscillations encode movement parameters like force and velocity and bind them in the generation of movement trajectories?

### Task-dependent modulations of ERD and ERS

The amplitude of M1 oscillations decreases in mu (8–13 Hz) and beta (13–30 Hz) bands prior to and during movement, followed by a post-movement rebound that exceeds the resting state level (Pfurtscheller and Lopes Da Silva, 1999). The time course of this event-related desynchronization (ERD) and synchronization (ERS) relates to several movement parameters. More forceful movements are accompanied by stronger mu and beta desynchronization (Stancak and Pfurtscheller, 1996; Stancak et al., 1997; Mima et al., 1999) and longer ERS (Stancak et al., 1997). The type of movement seems not to influence beta ERD but increases ERS when more muscle mass is involved, as demonstrated by stronger ERS for wrist compared to finger movement (Pfurtscheller et al., 1998) and shoulder compared to finger movement (Stancak et al., 2000). Movement duration has little to no effect on both ERD and ERS (Stancak and Pfurtscheller, 1996; Cassim et al., 2000). However, with increasing movement frequency the beta ERD becomes stronger and the ERS becomes less pronounced until the ERD and ERS peaks become almost indistinguishable (Toma et al., 2002; Houweling et al., 2010a). Also, ERD increases with complexity of sequential finger movements (Manganotti et al., 1998; Hummel et al., 2003).

Differences in mu and beta activation patterns imply that the two rhythms are independent, at least to a certain degree, and may thus have distinct functional relevance. During the early stages of movement execution especially mu desynchronization is spatially diffuse and only becomes somatotopically more focused when movements are sustained (Crone et al., 1998b). Compared to mu activation, beta suppression is more spatially focused (Crone et al., 1998b) and starts a little earlier, usually ending in a quicker and more noticeable ERS (Salmelin and Hari, 1994; Pfurtscheller et al., 1996; Alegre et al., 2003; Erbil and Ungan, 2007). Mu and beta modulations are also likely to originate from distinct sources located in postcentral (somatosensory) and precentral (motor) cortex, respectively (Salmelin and Hari, 1994; Salmelin et al., 1995; Cheyne et al., 2003; Ritter et al., 2009). Furthermore, mu synchronization and beta synchronization with motoneurons (MNs) in the spinal cord are clearly separate, as will be outlined below.

Event-related beta desynchronization already starts 2 s before movement in M1 contralateral to the active hand, and becomes bilateral during movement. As the pre-movement ERD is relatively unrelated to movement parameters, it has often been ascribed to an unspecific, general state of movement preparation (Neuper and Pfurtscheller, 2001). A lateralized ERD occurs when information is available which hand to move (Doyle et al., 2005; van Wijk et al., 2009). This is expressed by stronger power suppression over the hemisphere contralateral to the response hand, which is also accompanied by faster reaction times compared to a non-lateralized ERD. However, only in the final 100 ms the pre-movement preparation appears accompanied by increased corticospinal excitability (Chen et al., 1998; Leocani et al., 2000). Using transcranial magnetic stimulation, Chen et al. (1998) found a decrease in corticospinal excitability around the time of ERS. This is in keeping with the proposed movement inactivation function of ERS to recover from previous activation (Pfurtscheller et al., 1996).

Elevated beta activity is also present during tonic muscle contractions and disappears during movement. This led to the hypothesis that beta synchrony plays a role in stabilizing current motor output and, by the same token, in preventing initiation of new movements. A causal relation between increased beta oscillations and movement suppression was shown by Pogosyan et al. (2009) who entrained cortical beta oscillations with a transcranial alternating-current stimulation at 20 Hz. During episodes of stimulation, fast goal-directed movements were executed slower, while reaction time remained unaffected. This effect is specific to the beta band and does not occur for entrainment with 5 Hz oscillations. A similar slowing occurs when movements are initiated during spontaneous bursts of beta-frequency oscillations in the ongoing resting-state activity (Gilbertson et al., 2005). An up-regulation of beta synchrony is also used as an active mechanism to suppress unwanted motor output when subjects need to anticipate an upcoming perturbation of finger position (Androulidakis et al., 2007). Another example is the increased beta synchrony in a go/no-go task when movement needs to be withdrawn (Alegre et al., 2004; Zhang et al., 2008; Swann et al., 2009). These studies indicate that also an up-regulation of beta synchrony can be exploited in a behaviorally relevant way.

### Neuronal activity underlying population oscillations

In view of the binding-by-synchrony hypothesis, the just-discussed M1 power modulations in mu and beta bands may appear somewhat counterintuitive since synchronized firing in M1 disappears as soon as movements are executed. One may indeed expect a larger impact on MNs in the spinal cord when corticospinal inputs arrive simultaneously. Invasive recordings in the macaque motor cortex have been very helpful in tackling this issue because they highlight the spiking behavior of individual PTNs during oscillations of the M1 population.

Spikes of single neurons are phase-locked to the beta oscillations in the local field potentials (LFP) during periods in which beta oscillations are well pronounced (Murthy and Fetz, 1996b; Baker et al., 1997; Denker et al., 2007), though more variable relations have also been observed (Donoghue et al., 1998). While the spiking of individual PTNs may be only weakly coherent with the local LFP, summing over a population of PTNs strongly increases the phase locking to the LFP oscillations (Baker et al., 2003). More subtle synchronization between PTNs occurs between cell pairs that project to the same muscle fields as opposed to cell pairs with non-overlapping fields (Jackson et al., 2003). In particular the latter indicates that coordinated activity patterns are functionally organized and that synchronization may be important for individual neurons to cooperate.

The spiking activity of PTNs during movement execution, when both LFP beta power and synchrony with individual spiking neurons drop, strongly increases and shows a distinct inverse relation with LFP beta power (Baker et al., 2001; Spinks et al., 2008). The increase in firing rate reaches towards or into the gamma range (>30 Hz) (Baker et al., 2001; Grammont and Riehle, 2003). Although the inverse relation with LFP beta power emerges on a broad time span and does hence not reflect an instantaneous coupling (Spinks et al., 2008), it still suggests that an increased firing rate constitutes a prime mechanism to initiate changes in muscle activity. Interestingly, this may link to visual attention for which Bressler (1990) suggested that the attentional decrease in alpha and increase in gamma oscillations might both be distinct aspects of a shift in firing rate towards the gamma range, which might be a generic information-carrier across the cortex.

### Increased gamma band oscillations around movement onset

Invasive, single unit recordings are not the only means to measure an increase in firing rate of PTNs into the gamma range during movement execution. ECoG, EEG, and MEG have revealed bursts of gamma activity, peaking just before movement onset (Pfurtscheller et al., 1993), around movement onset (Pfurtscheller and Neuper, 1992), or slightly after it (Ohara et al., 2001; Pfurtscheller et al., 2003; Cheyne et al., 2008; Muthukumaraswamy, 2010), and they re-appear around movement offset (Szurhaj et al., 2005; Ball et al., 2008). Gamma bursts are only present in the hemisphere contralateral to movement where they have a focal somatotopic representation (Crone et al., 1998a; Szurhaj et al., 2005; Miller et al., 2007). Crone et al. (1998a) made a distinction between low (35–50 Hz) and high (75–100 Hz) gamma synchronization. Low-gamma ERS starts after movement onset and is sustained during almost the entire movement, whereas high-gamma ERS starts slightly earlier but is much more transient. Interestingly, the amplitude of the movement-related gamma increase may vary with movement direction, as observed in LFP (Rickert et al., 2005) and ECoG (Leuthardt et al., 2004; Ball et al., 2009) recordings. These studies provide support for a positive correlation between cortical gamma activity and movement execution. While tonic muscle contractions are promoted by beta oscillations, changes in motor output require gamma activity to arise.

## Cortico-spinal synchronization

Synchronized activity in M1 is modulated in the mu, beta, and gamma frequency bands during various stages of movement planning and execution. Are these oscillations also used to transfer motor commands to the spinal cord? Or framed differently, does synchronization occur between M1 and MN activity? And, if so, does it follow the same modulations as seen in the cortex or is it more than just a consequence of cortical drive?

### Rhythmic activity of motoneurons

A prerequisite for corticospinal synchronization is the presence of oscillatory MN activity. The firing of a single MN leads to a motor unit action potential (MUAP) that induces muscle fiber contraction. For an effective muscle contraction sequential stimulation is necessary to build up force. As a consequence, the firing rate of MNs determines motor unit output and ranges from 6 Hz in rest to 35 Hz during forceful isometric contractions. During faster contractions the firing rate may increase up to transient high-frequency bursts (80–120 Hz) as in the case of very rapid, ballistic movements (Freund, 1983). We note that single MUAPs can be recorded intramuscularly using wire-EMG, whereas surface-EMG captures summed MUAPs. The EMG signal is often rectified prior to estimating its spectral density to augment power related to MN firing rate (Myers et al., 2003). This information is contained primarily in frequencies below 40 Hz and the spectrum approaches the MUAP spectrum for higher frequencies. For these low frequencies, invasive recordings revealed synchronized spiking activity between pairs of motor units, which are likely caused by a common, central input (Farmer et al., 1993; Marsden et al., 1999; Kim et al., 2001; Kilner et al., 2002). This synchronization is not confined to motor units within the same muscle, but can also be observed between different muscles of the same limb (Kilner et al., 1999; Boonstra et al., 2008), as well as homologous muscles of the other limb (Boonstra et al., 2007b, 2008, 2009). With MN firing rate being such an important modulator of muscle activity, it might be entrained by cortical oscillations.

### Task-dependent modulations in corticospinal synchronization

M1 and contralateral muscle activity are weakly but significantly synchronized. This is most prominent in the beta frequency range during weak tonic muscle contractions (Conway et al., 1995; Salenius et al., 1997; Halliday et al., 1998; Gross et al., 2000) and disappears during movement (Baker et al., 1997; Kilner et al., 2000). Occasionally, significant mu synchronization can be detected but is less consistent over subjects (Salenius et al., 1997; Mima et al., 1999). Beta band corticospinal coherence is increased for isometric muscle contractions at moderate force levels (Witte et al., 2007; Chakarov et al., 2009). During (nearly) maximal force production the peak frequency shifts into the gamma range (Brown et al., 1998; Mima et al., 1999). Likewise, gamma corticospinal synchronization arises when rapid adjustments in force output in response to visual information are needed (Schoffelen et al., 2005; Andrykiewicz et al., 2007; Omlor et al., 2007). Furthermore, without visual input, gamma synchronization during phasic muscle contractions has been observed between ECoG and EMG (Marsden et al., 2000).

Both cortical power and corticospinal coherence in the beta band are enhanced when generated muscle force is kept constant around a certain target level with high precision (Kristeva et al., 2007; Witte et al., 2007). By contrast, coherence drops when attention is divided between the motor task and a simultaneously performed mental arithmetic task (Kristeva-Feige et al., 2002; Safri et al., 2007; Johnson et al., 2011). An up-regulation of corticospinal beta synchronization can be used to facilitate action selection by inhibiting the non-selected response hand (van Wijk et al., 2009). In sum, these results show that the role of beta oscillations in stabilizing motor output is not limited to the cortical level but extends to the spinal cord.

Remarkably, the strength of corticospinal synchronization depends on recent motor engagement. Larger digit displacement during a hold-ramp-hold task significantly increases beta corticospinal coherence (Riddle and Baker, 2006). Likewise, Omlor et al. (2011) showed that beta corticospinal coherence is strongly increased during isometric muscle contractions following a period of dynamic force production compared to after rest. Producing dynamic force that is unpredictable even further enhances coherence afterwards. The increase in coherence and the preceding cortical beta desynchronization were negatively correlated, while differences in accuracy of task performance were absent (Omlor et al., 2011). This suggests that after larger motor-related neural processing a stronger corticospinal synchronization is required to establish stable force production.

### Is corticospinal synchronization independent from modulations in cortical power?

The task-dependent modulations of corticospinal synchronization often coincide with similar changes in spectral power in motor cortex and in muscle activity. The question arises whether corticospinal synchronization is an independent, functional mechanism or merely a consequence (or by-product) of local synchronization. We note that if corticospinal synchronization is estimated using measures that explicitly depend on spectral power (e.g., coherence), the changes in power will certainly reflect on corticospinal synchronization estimates. Despite such technical caveats, there seems to be experimental support for the independence of power and corticospinal synchrony as they can be modulated separately.

In a study by Baker and Baker (2003), subjects performed a simple hold-ramp-hold task after administration of diazepam, a benzodiazepine that enhances inhibitory post-synaptic potentials via GABA_A_ receptors. While the amplitude of EEG beta power doubled in size, corticospinal coherence remained unaffected and even showed a slight decrease. In a subsequent study it was shown that the antiepileptic drug carbamazepine has an opposite effect: corticospinal synchronization is boosted while cortical power remains unaffected (Riddle et al., 2004). However, in this case the EMG power was also increased, which according to the authors might have occurred due to altered properties of muscle spindle afferents.

**Figure 1:**
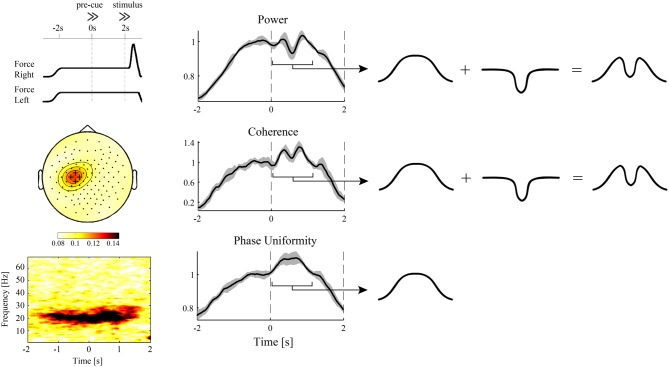
**Evidence for independent modulations in cortical power and corticospinal synchronization?** EEG and hand muscle EMG were recorded during a pre-cued reaction time task with either the left or right hand as response hand (van Wijk et al., 2009). During the interval between pre-cue and stimulus, which required static force production, significant beta band coherence between the EMG and sensors overlying the contralateral motor cortex was observed (**left panels**). The same electrodes showed a brief cue-related drop in spectral power (**middle panels**). Looking at the different time courses for power, corticospinal coherence, and corticospinal relative phase uniformity [also referred to as “phase-locking index” (Mardia, 1972; Lachaux et al., 1999)], the modulations in the pre-cue–stimulus interval could be decomposed into a slow, sustained modulation and the linearly superimposed brief cue-related drop (**right panels**). Remarkably, corticospinal phase uniformity only showed a sustained modulation, suggesting that the cue-related drop was not transferred to the spinal cord. On the other hand, corticospinal coherence explicitly depends on spectral power and was hence unable to discriminate between the different modulations in cortical and corticospinal synchronization. Alternatively, the cue-related drop might originate from nearby cortical sources that do not have projections to the spinal cord. For more details, see van Wijk et al. (2008).

Whether or not corticospinal synchronization can be modulated independently of spectral power under natural conditions remains to be seen (see Figure 1). Given the time-locked firing of PTNs during episodes of increased beta oscillations, one may expect a strong dependence of corticospinal synchronization on cortical power. Then, synchronization readily occurs when MNs respond to the oscillatory input they receive. As outlined in more detail below, however, we consider this view an oversimplification because the contribution of afferent pathways is ignored.

### Mere entrainment or interaction via afferents?

Corticospinal synchronization can emerge through a uni-directional coupling from cortex to spinal cord. Such synchronization implies that MN activity is entrained by efferent cortical activity. On the other hand, cortical motor areas do receive strong input from the periphery either via the somatosensory cortex or projections from thalamic nuclei (Lemon and Vanderburg, 1979). This information may be used to adjust the cortical drive depending on the current motor state. Hints at a more involved mechanism of corticospinal synchronization can be obtained by estimating the according phase delay. In the case of a sole efferent drive, a fixed delay should be present that corresponds to the time it takes for an action potential to travel from the cortex to its target MN. This means that the phase delay would increase linearly with frequency. Although some experimental findings favor this view (Gross et al., 2000), the phase delay in question has been found to be too short compared to physiological conduction times (Salenius et al., 1997; Brown et al., 1998; Mima et al., 2000b; Riddle and Baker, 2005; Houweling et al., 2010b), to contain both leading and lagging features relative to cortical activity (Muthuraman et al., 2008; Williams et al., 2009), or to be constant (Halliday et al., 1998; Riddle and Baker, 2005).

This mishmash is not the only reason that to date there is no real consensus about the contribution of afferent pathways to corticospinal synchronization. Support for a role of afferent feedback comes from a study by Riddle and Baker (2005), which showed that increasing corticospinal conduction times induced via arm cooling resulted in a decrease in corticospinal coherence that cannot be explained by a change in efferent drive only. The reported additional time delay between cortex and spinal cord due to arm cooling was twice the conduction time in one direction, suggesting that bi-directional pathways contributed to the generation of coherence. In contrast, Gerloff et al. (2006) ruled out any contribution from sensory pathways in a patient population with interhemispheric reorganization of motor areas due to pyramidal tract lesions. In these patients, the other hemisphere had substituted the function of M1 in the affected hemisphere, but not that of S1. Due to the clear spatial separation between S1 and M1, the origin of corticospinal coherence could be pinpointed to M1.

The direction of information flow between cortex and spinal cord can also be inferred using directionality measures like Granger causality and the directed transfer function (for alternative measures see, e.g., Nolte et al., 2008; Muskulus et al., 2009). Mima et al. (2001) found the efferent coherence from cortex to spinal cord to be larger than in the opposite direction, although afferent coherence was still significant (Tsujimoto et al., 2009). Witham et al. (2011) even reported a stronger coherence in the afferent direction in a subpopulation of subjects. Gross et al. (2002) looked at the directionality of synchronization between sensorimotor cortex and the EMG for the 6–9 Hz discontinuities that can be observed during smooth movements. Using source analysis, they showed that the sign of directionality reversed around the central sulcus, with predominantly efferent coupling for the motor cortex and afferent coupling for the somatosensory cortex. Oscillatory activity also appears to be present in the firing of group Ia afferents that carry information about changes in muscle fiber length (Baker et al., 2006). Brovelli et al. (2004) reported directional beta band coherence from somatosensory cortex to primary motor cortex. These mechanisms could in fact serve to close the feedback loop to the motor cortex and allow for sensorimotor integration. If so, corticospinal synchronization constitutes an intricate mechanism for improving motor performance by means of a bi-directional coupling between cortex and spinal cord rather than a mere reflection of an efferent drive.

## Cortico-cortical synchronization

Obviously, contralateral primary motor cortex is not the only brain region that is involved in motor control. Depending on the task at hand, premotor, parietal, subcortical, and cerebellar areas are active in both contra- and ipsilateral hemispheres. Given the abundant evidence for synchronization between motor cortex and spinal cord, it can be expected that similar mechanisms also mediate communication between these areas. Studying these mechanisms is a challenge: whereas the distance between motor cortex and limb muscles is large enough to avoid interference of neural activity in the recorded signals, the activity of different brain regions is more difficult to discriminate with non-invasive techniques.

### Non-invasive studies

Several EEG and MEG studies have shown cortico-cortical synchronization patterns indicating distinct forms of neural communication. Beta band coherence between motor and visual cortex is increased when subjects perform a visuomotor tracking task and decreased when the visual stimulus is a mere distractor (Classen et al., 1998). Interhemispheric beta band coherence between motor areas arises during both unimanual and bimanual rhythmic movements (Mima et al., 2000a; Gross et al., 2005), and increases with task complexity (Gerloff et al., 1998; Manganotti et al., 1998; Gross et al., 2005). Conflicting results have been found regarding the effect of movement rate on interhemispheric coherence (Serrien and Brown, 2002; Toma et al., 2002). In line with the involvement of SMA in self-initiated movements, coherence between sensorimotor cortex and mesial premotor areas is larger for internally compared to externally paced movements (Gerloff et al., 1998; Serrien, 2008). During auditory-cued finger tapping, Pollok et al. (2005) found an extensive network of activated brain regions including M1, SMA, premotor cortex, posterior parietal cortex, auditory cortex, thalamus, and cerebellum. Across these areas significant coherence could be observed, mainly in the alpha band. In a similar study, inter-cerebellar coherence was found to be increased for bimanual synchronous finger tapping (Pollok et al., 2007).

Learning a complex motor task can alter cortico-cortical synchronization. Andres et al. (1999) trained subjects to integrate two unimanual tapping sequences into a new, bimanual sequence. Initially, interhemispheric mu and beta band coherence between sensorimotor areas increased during the training period. After successful learning of the bimanual sequence the interhemispheric coherence was decreased again. Hence, when a bimanual skill has been acquired, less interhemispheric interaction is needed to perform the task. Similar decreases in interhemispheric coherence were obtained by Serrien and Brown (2003), along with a strong initial increase in interhemispheric gamma coherence between pre-frontal areas. The latter may reflect an increased cognitive demand needed to learn a novel motor task. Also perceptual learning of a tactile discrimination task may alter functional connectivity between sensorimotor areas (Freyer et al., 2012). In addition to cortical interregional synchronization, motor learning is also associated with changes in spectral power (Boonstra et al., 2007a; Houweling et al., 2008) and synchronization between motor cortex and spinal cord (Houweling et al., 2010b). These findings suggest that neural activation patterns do not only relate to movement execution but also to one's familiarity with or expertise in the motor task. That is, movement-related synchronization patterns are not permanently configured but can be “reshaped” dynamically with experience.

### Synchronization or volume conduction?

**Figure 2:**
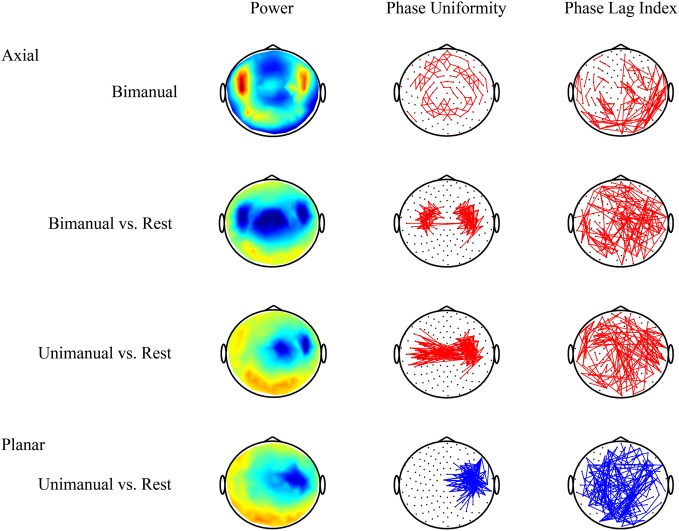
**Volume conduction complicates the interpretation of connectivity patterns estimated from MEG or EEG recordings.** Data were recorded using MEG with axial gradiometers and group results for the beta band (20–25 Hz) are shown (see also, van Wijk et al., 2012). **Top row**: activity during bimanual force production alone is not very informative. Neighboring sensors show strong relative phase uniformity as they pick up activity of common sources. **Second row**: contrasting movement with resting state yields the characteristic movement-related power decrease over motor cortices. In addition, two clusters of increased connectivity are evident that seem to be located in between the locations with largest power suppression. However, the phase lag index reveals that all pair-wise connections with non-zero or non-pi phase difference are distributed randomly over the scalp. Hence, one cannot rule out that the increased local connectivity is caused by volume conduction. **Third row**: for unimanual movement there even seems to be increased interhemispheric coupling. But again it is difficult to discern whether these connections express true in-phase synchronization. **Bottom row**: by contrast, a transformation to planar gradients reveals a strong decrease in connectivity overlying motor areas that coincides with a drop in power. This means that, due to less beta activity, the estimated relative phase uniformity between neighboring sensors is weaker compared to resting state. Increases in power and connectivity are indicated in red, decreases in blue. Only the strongest connections are shown.

A major obstacle in studying cortico-cortical synchronization using non-invasive recording techniques is the presence of volume conduction. Because of the distance between the recording sites and the cortical tissue, activity of a single dipole source will be picked up by multiple electrodes/sensors. Furthermore, the conductivity properties of different tissues between cortex and recording sites lead to a blurring of electric potentials on the scalp. The latter affects EEG, whereas the distance between sensors and cortex is larger for MEG. As a result, the activity of neighboring recording sites is highly coherent with a zero-lag phase difference. Due to this effect, Srinivasan et al. (2007) estimated that the coherence between EEG electrodes separated by less than 10 cm is considerably elevated, as well as a small effect for widely separated electrodes (>20 cm). For MEG, substantial field-spread effects on the coherence estimates occur for sensors separated by less than 15 cm. As two underlying sources may show genuine in-phase locking, it becomes very difficult if not impossible to distinguish true in-phase synchronization from volume conduction artifacts (see Figure 2).

Another consequence of volume conduction is a bias in both amplitude and relative phase estimates of two sources, as demonstrated by Tognoli and Kelso (2009). Due to the superposition of activity from multiple sources at the sensor level, the amplitude of anti-phase coupled oscillators is diminished and amplified for in-phase coupled oscillators. Moreover, the relative phase between oscillators at the source level coupled with a phase lag other than zero will appear more in-phase synchronized at the sensor level. This implies that the synchronization patterns observed in EEG/MEG recordings might not reflect the amplitude and phase coupling between sources. The authors further show that a “waxing and waning” pattern, as can be observed in occipital alpha activity, can result from two non-interacting oscillators that periodically approach an in- or anti-phase phase relation. On the whole, the superposition of source activity complicates interpretation of non-invasively observed synchronization patterns to say the least.

Attempts to circumvent the volume conduction problem involve the use of different measures of functional connectivity that either look at phase synchronization that is not centered around zero or ±180° (Nolte et al., 2004; Stam et al., 2007), or try to decompose the sensor signal into separate sources while preserving the interactions among them (Meinecke et al., 2005; Gomez-Herrero et al., 2008; Marzetti et al., 2008). Although these methods are effective in diminishing volume conduction effects (the first two are in fact fully invariant against zero-lag volume conduction), they are also very conservative as all true in- or anti-phase synchronization is ignored. Applying inverse methods to estimate synchronization on the source level does not solve the problem (Schoffelen and Gross, 2009). Probably the best way to avoid spurious synchronization from volume conduction effects is to record brain activity invasively.

### Intracranial studies

LFP recordings in the macaque revealed episodes of synchronous beta oscillations. Murthy and Fetz (1996a) reported synchronous oscillations between sites within motor areas, between pre- and post-central sites and occasionally also between bilateral motor areas. Oscillations occurred more often and were stronger during exploratory movements compared to rest but seemed unrelated to specific movement phases. More systematic modulations in relation to movement have also been found for intra- and inter-hemispheric synchronization. Synchronization within and between primary motor cortex and premotor areas is present in the time period before movement and ceases prior to movement initiation (Sanes and Donoghue, 1993). The activity of bilateral M1s shows a larger correlation around movement onset of synchronous bimanual movements compared to asynchronous bimanual and unimanual movements (de Oliveira et al., 2001). In contrast, the period during movement execution is characterized by a decrease in interhemispheric correlation. In addition, an in-phase synchronization in the 10–40 Hz range can be observed during isometric contractions between deep cerebellar nuclei and bilateral M1s (Soteropoulos and Baker, 2006).

In humans, Ohara et al. (2001) found an increased coherence for frequencies <50 Hz around movement onset between S1 and M1 within the same hemisphere. They also observed increases in predominantly mu band coherence between S1/M1 and SMA that started almost 1 s before movement onset and peaked after movement. The time course of coherence, however, did not fully agree with changes in spectral power, which started earlier and were maximal for higher frequencies. The data were obtained from ECoG recordings in epilepsy patients during self-paced brisk finger extension. For the majority of subjects, synchronized regions showed a phase lag close to zero.

One is tempted to conclude that true in-phase synchrony is present across the cortex and is modulated in service of motor function. If so, the synchrony should have the capacity to carry information relevant for motor performance and/or its control. In fact, first evidence for information carried in synchronized neural activity comes from the study of Stark et al. (2008), who recorded single- and multi-unit activity in premotor areas of two monkeys. The monkeys performed a prehension task with a short delay period within which information about reach direction and grip type (precision or opposition) had to be memorized. During this period, most single and multi-unit activity recorded showed a preference for reach direction and, to a lesser extent, grip type but not so much for their combination. However, pair-wise cross-correlation between multi-unit activity revealed specificity to the combination of reach direction and grip type. This information was not apparent from single-unit recordings but only in the averaged activity of small groups of neurons and the synchronization did not have an oscillatory nature.

## Abnormal synchronization patterns in motor dysfunction

Altered synchronization patterns due to disease states that give rise to motor impairment can provide important insight into activation patterns underlying healthy motor functioning. We here sketch some seminal examples.

### Parkinson's disease

The bradykinesia seen in Parkinson's disease is intimately related to excessive beta oscillations in the basal ganglia (for a review, see Brown, 2007; Hammond et al., 2007). Both treatment with dopaminergic drugs and high frequency deep brain stimulation (DBS) in the subthalamic nucleus (STN) or globus pallidus leads to suppression of the excessive beta activity (Brown et al., 2001, 2004; Priori et al., 2004; Wingeier et al., 2006). Moreover, the degree of suppression in STN correlates with clinical improvement (Kuhn et al., 2006, 2008, 2009; Weinberger et al., 2006; Ray et al., 2008). The effect of DBS depends on the frequency of stimulation. In contrast to high frequency stimulation (100–180 Hz), stimulation of the STN with 20 Hz slows movement execution (Chen et al., 2007), suggesting a causal relation between the excessive oscillations and bradykinesia. Interestingly, the phase pattern of stimulation also modifies effects of DBS as phase resetting is known for its capacity to desynchronize oscillatory neural populations (Tass, 1999, 2002; Hauptmann et al., 2009). Although resting state oscillatory activity in M1 seems hardly affected in Parkinson's disease (Brown, 2007; but see Goldberg et al., 2002; Vardy et al., 2011), movement-related ERD is delayed and lower in amplitude compared to healthy controls while ERS is diminished (Defebvre et al., 1998; Wang et al., 1999; Magnani et al., 2002; Devos et al., 2003a,b). In addition, corticospinal beta synchronization levels are reduced in untreated state, while corticospinal synchronization for lower frequencies associated with tremor is enhanced (Salenius et al., 2002). Cortico-cortical beta synchronization is elevated in untreated state as well (Silberstein et al., 2005). Levodopa administration and DBS also restores these patterns towards normal levels.

### Essential tremor

One of the most prevalent movement disorders is essential tremor (Lorenz and Deuschl, 2007). It is characterized by a bilateral action tremor in arms and hands of around 6–12 Hz. Its origin lies in the central nervous system but cannot be pinpointed to a single area. Instead, a network of regions is believed to be involved in its generation, including the inferior olive, cerebellum, thalamus, and motor cortex (Raethjen and Deuschl, 2012). Schnitzler et al. (2009) reported coherent activity among these regions at the tremor frequency and its harmonics. This activity is propagated to the spinal cord as revealed by significant corticospinal coherence at the tremor frequency (Hellwig et al., 2001). However, despite the tremor being quite steady over time, the corticospinal coherence appears intermittently (Raethjen et al., 2007). This may hint at the involvement of other (sub)cortical regions within the identified network in the propagation of oscillatory activity to the spinal cord. It has been submitted that abnormal synchronization patterns within similar networks might also underlie other types of tremors (Schnitzler et al., 2009). In healthy subjects, one can observe a physiological tremor in the same frequency range as the essential tremor but it typically has much smaller EMG amplitude. Physiological tremor occurs during smooth continuous movements and appears coherent with contralateral M1 (Gross et al., 2002). A reason that these oscillations are only small under normal circumstances might be that circuits in the spinal cord counteract the descending 10 Hz drive from the brain through phase cancellation (Williams et al., 2010).

### Mirror movements

Unilateral movements may evoke unintended muscle activity of the passive limb. In healthy subjects, mirror movements are more frequently seen in young children, whose corpus callosum is not yet fully myelinated, and in elderly, who undergo atrophy of white matter pathways. In both cases they are expressed more when fatigued (Duque et al., 2005). The cause for the emergence of mirror movements might be either uncrossed ipsilateral corticospinal pathways or improper suppression of interhemispheric crosstalk (Shibasaki and Nagae, 1984; Britton et al., 1991; Daffertshofer et al., 1999, 2005). Still, the contribution of ipsilateral and bilateral activation pathways is under debate (for review see Addamo et al., 2007). In line with the bilateral activation theory, Huo et al. (2011) showed ipsilateral gamma synchronization around unimanual movement onset in children, the incidence of which declined with age. Such ipsilateral activity is absent in adults. Mirror movements may also occur under pathological conditions and they are particularly pronounced in the case of callosal damage (Dennis, 1976; Bonzano et al., 2008). A case study of a patient suffering from congenital mirror movements showed significant corticospinal coherence for the passive hand with the ipsilateral but not with the contralateral motor cortex (Pohja et al., 2002). Equivalent results were found for two patients with X-linked Kallmann's syndrome, a third patient showed coherence with bilateral motor cortices (Farmer et al., 2004). In these cases, mirror movements are likely to arise from uncrossed pathways. These examples demonstrate that the origin of mirror movements could be inferred by looking at synchronization patterns.

## Discussion

Causal relations between encephalographic signals and motor behavior are difficult to demonstrate and synchronization can be epiphenomenal to power changes for a variety of reasons. There are, however, several findings that refute the latter possibility. For instance, the aforementioned study by Pogosyan et al. (2009) showed a slowing of movements with experimentally entrained beta oscillations. Also, DBS studies in patients with Parkinson's disease revealed that stimulation at 20 Hz deteriorates motor function (Chen et al., 2007), whereas higher stimulation frequencies yield a break-down of abnormal synchronization patterns and, in consequence, result in clinical improvement (Kuhn et al., 2006, 2008, 2009; Weinberger et al., 2006; Ray et al., 2008). Admittedly, corticospinal synchronization values are typically low albeit significant and therefore seem to be of little functional relevance. Modeling work suggests that the impact of corticospinal synchronization might be masked by the non-linear properties of MNs (Williams and Baker, 2009; Negro and Farina, 2011). Also, a true effect of synchronization often becomes only apparent on a population level as the activity of single neurons might be only weakly correlated to the ensemble average (Baker et al., 2003). Oscillatory activity in single neurons can be difficult to detect but it is apparent when summing over multiple neurons (Donoghue et al., 1998). We note, however, that the neurons of isolated pair-wise interactions are always embedded in a network of numerous interacting neurons, i.e., a neural population. It is therefore fair to assume that a functional role of synchronization lies in its capacity for *population coding* rather than its impact on single neuron activity.

We further note that the aforementioned representation of motor programs via (temporally bound) cell assemblies (Wickens et al., 1994; Hommel, 2004) is in line with the concept of population coding. Assemblies may be formed across spatially distributed sites that functionally contribute to the same movement feature. They form small networks that show coordinated (sequential) activity when their movement feature is expressed. Interestingly, Brown and Marsden (2001) reported coherence between the cortical representations of wrist flexion and extension when they act in unison, compared to no such binding when the muscle contractions were executed separately. Neurons may be part of multiple assemblies encoding different movement features and may shape new assemblies during motor learning.

Despite the omnipresence of synchronized neural activity across the brain, some skepticism about its role as information carrier persists (Shadlen and Movshon, 1999). In many cases, it is difficult to distinguish synchronization from *rate coding*, in which the discharge frequency of neuronal populations conveys task-related information. An example of rate coding is the so-called population vector for movement direction in M1 (Georgopoulos et al., 1986). In fact, synchronization and rate coding often correlate. The reason for this might be trivial as a higher spike rate increases the probability of detecting coinciding spikes that will be labeled as synchronized. This confounder should be taken into account in determining significance of synchronization though temporal variations in spike rate do complicate a clear-cut separation of true synchronization and spike rate modulations. There is even experimental evidence in favor of a role of rate covariation, rather than synchronization, as a binding mechanism in the visual system (Nirenberg et al., 2001; Golledge et al., 2003; Roelfsema et al., 2004). Also in the motor cortex synchronization has been found to provide no additional information on movement direction compared to spike frequency (Oram et al., 2001). Evidently, synchronization between neuronal populations might not be the only way in which brain regions communicate.

Importantly, rate coding and synchronization are not mutually exclusive mechanisms but may operate cooperatively. Synchronous discharges between cell pairs in M1 may show directional specificity different from their directional tuning curves based on firing rate (Hatsopoulos et al., 1998). Also, synchronization may arise both with and without alterations in neural firing rate. Grammont and Riehle (2003) showed that the time course of spike coincidence and firing rate display different modulations during movement preparation and execution. Episodes of increased spike coincidence mainly occur at the end of the preparation period, without an obvious relation to firing rate. During movement execution however, synchronization drops while firing rate strongly increases. Moreover, synchronization that is not accompanied by alterations in firing rate is associated with the processing of internal cognitive events and the concurrent modulation of synchronization and firing rate with external events (Riehle et al., 1997). Hence spike timing might not act alone in transferring information but might have an ally in firing rate modulations.

Inter-regional synchronization is usually studied using linear measures of interdependencies like cross-correlation, coherence, or iso-frequency (1:1) phase locking. However, given the complex behavior of small neural circuits, additional information might be contained in non-linear coupling, also on larger scales. On the single neuron level, more complex types of correlations than spike coincidence, like long-range correlations and common patterns of spikes might occur that are ignored with commonly used synchronization measures (Latham and Nirenberg, 2005). On a population level, coupling between frequency bands might occur. Measures of non-linear-interdependencies are more adequate in capturing these types of interactions. This includes cross-frequency (n:m) phase locking (Tass et al., 1998), bi-coherence, synchronization in state-space and information theoretic measures like mutual information (for an overview of commonly used linear and non-linear measures see Pereda et al., 2005). Only few studies have investigated non-linear interactions in the motor system (Calmels et al., 2008; Darvas et al., 2009; Muskulus et al., 2009; Chen et al., 2010; Jin et al., 2010). Interestingly, Chen et al. (2008) were able to detect interactions between bilateral motor areas and between muscle activity and ipsilateral motor cortex using cross mutual information but not with standard coherence analysis. By looking at non-linear brain interactions, future studies will certainly advance our understanding of neural activity accompanying motor control.

## Conclusion

Synchronization seems to be an integral part of the neural control of movement. Throughout the motor system, regional and inter-regional synchronization patterns display task-related modulations. In order to understand how these activity patterns may possibly yield motor behavior, it is necessary to employ multiple recording techniques, data analysis tools, and modeling approaches. The paradoxical beta power decrease in M1 during movement can only be explained by the increased firing rates of the PTNs, hence boosting output to the spinal cord. This also underscores that neural communication is not solely achieved by synchronization. Rate coding and spike timing act conjointly during motor control. It is this combination that is pivotal for interpreting synchronization patterns.

### Conflict of interest statement

The authors declare that the research was conducted in the absence of any commercial or financial relationships that could be construed as a potential conflict of interest.

## References

[B1] Addamo P. K. Farrow M. Hoy K. E. Bradshaw J. L. Georgiou-Karistianis N. (2007). The effects of age and attention on motor overflow production - a review. Brain Res. Rev. 54, 189–204 10.1016/j.brainresrev.2007.01.004 17300842

[B2] Adrian E. D. Matthews B. H. C. (1934a). The berger rhythm potential changes from the occipital lobes in man. Brain 57, 355–385 20058345 10.1093/brain/awp324

[B3] Adrian E. D. Matthews B. H. C. (1934b). The interpretation of potential waves in the cortex. J. Physiol. 81, 440–471 16994555 10.1113/jphysiol.1934.sp003147 PMC1394145

[B4] Adrian E. D. Yamagiwa K. (1935). The origin of the berger rhythm. Brain 58, 317–351

[B5] Aertsen A. Vaadia E. Abeles M. Ahissar E. Bergman H. Karmon B. Lavner Y. Margalit E. Nelken I. Rotter S. (1991). Neural interactions in the frontal cortex of a behaving monkey: signs of dependence on stimulus context and behavioural state. J. Hirnforsch. 32, 735–743 1821420

[B6] Alegre M. Gurtubay I. G. Labarga A. Iriarte J. Valencia M. Artieda J. (2004). Frontal and central oscillatory changes related to different aspects of the motor process: a study in go/no-go paradigms. Exp. Brain Res. 159, 14–22 10.1007/s00221-004-1928-8 15480586

[B7] Alegre M. Labarga A. Gurtubay I. G. Iriarte J. Malanda A. Artieda J. (2003). Movement-related changes in cortical oscillatory activity in ballistic, sustained and negative movements. Exp. Brain Res. 148, 17–25 10.1007/s00221-002-1255-x 12478393

[B8] Andres F. G. Mima T. Schulman A. E. Dichgans J. Hallett M. Gerloff C. (1999). Functional coupling of human cortical sensorimotor areas during bimanual skill acquisition. Brain 122, 855–870 10.1093/brain/122.5.855 10355671

[B9] Androulidakis A. G. Doyle L. M. F. Yarrow K. Litvak V. Gilbertson T. P. Brown P. (2007). Anticipatory changes in beta synchrony in the human corticospinal system and associated improvements in task performance. Eur. J. Neurosci. 25, 3758–3765 10.1111/j.1460-9568.2007.05620.x 17610595

[B10] Andrykiewicz A. Patino L. Naranjo J. R. Witte M. Hepp-Reymond M. C. Kristeva R. (2007). Corticomuscular synchronization with small and large dynamic force output. BMC Neurosci. 8, 101 10.1186/1471-2202-8-101 18042289 PMC2245954

[B11] Asanuma H. Zarzecki P. Jankowska E. Hongo T. Marcus S. (1979). Projection of individual pyramidal tract neurons to lumbar motor nuclei of the monkey. Exp. Brain Res. 34, 73–89 103742 10.1007/BF00238342

[B12] Baker M. R. Baker S. N. (2003). The effect of diazepam on motor cortical oscillations and corticomuscular coherence studied in man. J. Physiol. (Lond.) 546, 931–942 10.1113/jphysiol.2002.029553 12563016 PMC2342588

[B13] Baker S. N. (2007). Oscillatory interactions between sensorimotor cortex and the periphery. Curr. Opin. Neurobiol. 17, 649–655 10.1016/j.conb.2008.01.007 18339546 PMC2428102

[B14] Baker S. N. Chiu M. Fetz E. E. (2006). Afferent encoding of central oscillations in the monkey arm. J. Neurophysiol. 95, 3904–3910 10.1152/jn.01106.2005 16709725

[B15] Baker S. N. Olivier E. Lemon R. N. (1997). Coherent oscillations in monkey motor cortex and hand muscle EMG show task-dependent modulation. J. Physiol. (Lond.) 501, 225–241 9175005 10.1111/j.1469-7793.1997.225bo.x PMC1159515

[B16] Baker S. N. Pinches E. M. Lemon R. N. (2003). Synchronization in monkey motor cortex during a precision grip task. II. Effect of oscillatory activity on corticospinal output. J. Neurophysiol. 89, 1941–1953 10.1152/jn.00832.2002 12686573

[B17] Baker S. N. Spinks R. Jackson A. Lemon R. N. (2001). Synchronization in monkey motor cortex during a precision grip task. I. Task-dependent modulation in single-unit synchrony. J. Neurophysiol. 85, 869–885 11160519 10.1152/jn.2001.85.2.869

[B18] Ball T. Demandt E. Mutschler I. Neitzel E. Mehring C. Vogt K. Aertsen A. Schulze-Bonhage A. (2008). Movement related activity in the high gamma range of the human EEG. Neuroimage 41, 302–310 10.1016/j.neuroimage.2008.02.032 18424182

[B19] Ball T. Schulze-Bonhage A. Aertsen A. Mehring C. (2009). Differential representation of arm movement direction in relation to cortical anatomy and function. J. Neural Eng. 6, 016006 10.1088/1741-2560/6/1/016006 19155551

[B20] Basar E. (1998). Brain functions and oscillations, in Brain Oscillations, Principles and Approaches, Vol. 1 Berlin: Springer

[B21] Berger H. (1929). Über das elektroenkephalogramm des menschen. Arch. Psychiatr. Nervenkr. 87, 527–570

[B22] Bonzano L. Tacchino A. Roccatagliata L. Abbruzzese G. Mancardi G. L. Bove M. (2008). Callosal contributions to simultaneous bimanual finger movements. J. Neurosci. 28, 3227–3233 10.1523/JNEUROSCI.4076-07.2008 18354026 PMC6670695

[B23] Boonstra T. W. Daffertshofer A. Breakspear M. Beek P. J. (2007a). Multivariate time-frequency analysis of electromagnetic brain activity during bimanual motor learning. Neuroimage 36, 370–377 10.1016/j.neuroimage.2007.03.012 17462913

[B24] Boonstra T. W. Daffertshofer A. van As E. van der Vlugt S. Beek P. J. (2007b). Bilateral motor unit synchronization is functionally organized. Exp. Brain Res. 178, 79–88 10.1007/s00221-006-0713-2 17109111

[B25] Boonstra T. W. Daffertshofer A. van Ditshuizen J. C. van den Heuvel M. R. C. Hofman C. Willigenburg N. W. Beek P. J. (2008). Fatigue-related changes in motor-unit synchronization of quadriceps muscles within and across legs. J. Electromyogr. Kinesiol. 18, 717–731 10.1016/j.jelekin.2007.03.005 17462912

[B26] Boonstra T. W. van Wijk B. C. M. Praamstra P. Daffertshofer A. (2009). Corticomuscular and bilateral EMG coherence reflect distinct aspects of neural synchronization. Neurosci. Lett. 463, 17–21 10.1016/j.neulet.2009.07.043 19619608

[B27] Bressler S. L. (1990). The gamma wave: a cortical information carrier? Trends Neurosci. 13, 161–162 10.1016/0166-2236(90)90039-D 1693231

[B28] Britton T. C. Meyer B. U. Benecke R. (1991). Central motor pathways in patients with mirror movements. J. Neurol. Neurosurg. Psychiatry 54, 505–510 1880512 10.1136/jnnp.54.6.505 PMC488588

[B29] Brovelli A. Ding M. Z. Ledberg A. Chen Y. H. Nakamura R. Bressler S. L. (2004). Beta oscillations in a large-scale sensorimotor cortical network: directional influences revealed by Granger causality. Proc. Natl. Acad. Sci. U.S.A. 101, 9849–9854 10.1073/pnas.0308538101 15210971 PMC470781

[B30] Brown P. (2000). Cortical drives to human muscle: the Piper and related rhythms. Prog. Neurobiol. 60, 97–108 10.1016/S0301-0082(99)00029-5 10622378

[B31] Brown P. (2007). Abnormal oscillatory synchronisation in the motor system leads to impaired movement. Curr. Opin. Neurobiol. 17, 656–664 10.1016/j.conb.2007.12.001 18221864

[B32] Brown P. Marsden J. F. (2001). Cortical network resonance and motor activity in humans. Neuroscientist 7, 518–527 10.1177/107385840100700608 11765129

[B33] Brown P. Mazzone P. Oliviero A. Altibrandi M. G. Pilato F. Tonali P. A. Di Lazzaro V. (2004). Effects of stimulation of the subthalamic area on oscillatory pallidal activity in Parkinson's disease. Exp. Neurol. 188, 480–490 10.1016/j.expneurol.2004.05.009 15246847

[B34] Brown P. Oliviero A. Mazzone P. Insola A. Tonali P. Di Lazzaro V. (2001). Dopamine dependency of oscillations between subthalamic nucleus and pallidum in Parkinson's disease. J. Neurosci. 21, 1033–1038 11157088 10.1523/JNEUROSCI.21-03-01033.2001 PMC6762327

[B35] Brown P. Salenius S. Rothwell J. C. Hari R. (1998). Cortical correlate of the Piper rhythm in humans. J. Neurophysiol. 80, 2911–2917 9862895 10.1152/jn.1998.80.6.2911

[B36] Buzsáki G. (2006). Rhythms of the Brain. Oxford: Oxford University Press

[B37] Calmels C. Hars M. Holmes P. Jarry G. Stam C. J. (2008). Non-linear EEG synchronization during observation and execution of simple and complex sequential finger movements. Exp. Brain Res. 190, 389–400 10.1007/s00221-008-1480-z 18604526

[B38] Cassim F. Szurhaj W. Sediri H. Devos D. Bourriez J. L. Poirot I. Derambure P. Defebvre L. Guieu J. D. (2000). Brief and sustained movements: differences in event-related (de)synchronization (ERD/ERS) patterns. Clin. Neurophysiol. 111, 2032–2039 11068239 10.1016/s1388-2457(00)00455-7

[B39] Chakarov V. Naranjo J. R. Schulte-Monting J. Omlor W. Huethe F. Kristeva R. (2009). Beta-range EEG-EMG coherence with isometric compensation for increasing modulated low-level forces. J. Neurophysiol. 102, 1115–1120 10.1152/jn.91095.2008 19458142

[B40] Chatrian G. E. Petersen M. C. Lazarte J. A. (1959). The blocking of the rolandic wicket rhythm and some central changes related to movement. Electroencephalogr. Clin. Neurophysiol. 11, 497–510 13663823 10.1016/0013-4694(59)90048-3

[B41] Chen C. C. Hsieh J. C. Wu Y. Z. Lee P. L. Chen S. S. Niddam D. M. Yeh T. C. Wu Y. T. (2008). Mutual-information-based approach for neural connectivity during self-paced finger lifting task. Hum. Brain Mapp. 29, 265–280 10.1002/hbm.20386 17394211 PMC6871222

[B42] Chen C. C. Kilner J. M. Friston K. J. Kiebel S. J. Jolly R. K. Ward N. S. (2010). Nonlinear coupling in the human motor system. J. Neurosci. 30, 8393–8399 10.1523/JNEUROSCI.1194-09.2010 20573886 PMC2923068

[B43] Chen C. C. Litvak V. Gilbertson T. Kuhn A. Lu C. S. Lee S. T. Tsai C. H. Tisch S. Limousin P. Hariz M. Brown P. (2007). Excessive synchronization of basal ganglia neurons at 20 Hz slows movement in Parkinson's disease. Exp. Neurol. 205, 214–221 10.1016/j.expneurol.2007.01.027 17335810

[B44] Chen R. Yaseen Z. Cohen L. G. Hallett M. (1998). Time course of corticospinal excitability in reaction time and self-paced movements. Ann. Neurol. 44, 317–325 10.1002/ana.410440306 9749597

[B45] Cheyne D. Bells S. Ferrari P. Gaetz W. Bostan A. C. (2008). Self-paced movements induce high-frequency gamma oscillations in primary motor cortex. Neuroimage 42, 332–342 10.1016/j.neuroimage.2008.04.178 18511304

[B46] Cheyne D. L. Gaetz W. Garnero L. Lachaux J. P. Ducorps A. Schwartz D. Varela F. J. (2003). Neuromagnetic imaging of cortical oscillations accompanying tactile stimulation. Cogn. Brain Res. 17, 599–611 10.1016/S0926-6410(03)00173-3 14561448

[B47] Classen J. Gerloff C. Honda M. Hallett M. (1998). Integrative visuomotor behavior is associated with interregionally coherent oscillations in the human brain. J. Neurophysiol. 79, 1567–1573 9497432 10.1152/jn.1998.79.3.1567

[B48] Cobb S. R. Buhl E. H. Halasy K. Paulsen O. Somogyi P. (1995). Synchronization of neuronal-activity in hippocampus by individual gabaergic interneurons. Nature 378, 75–78 10.1038/378075a0 7477292

[B49] Conway B. A. Halliday D. M. Farmer S. F. Shahani U. Maas P. Weir A. I. Rosenberg J. R. (1995). Synchronization between motor cortex and spinal motoneuronal pool during the performance of a maintained motor task in man. J. Physiol. (Lond.) 489, 917–924 8788955 10.1113/jphysiol.1995.sp021104 PMC1156860

[B50] Crone N. E. Miglioretti D. L. Gordon B. Lesser R. P. (1998a). Functional mapping of human sensorimotor cortex with electrocorticographic spectral analysis—II. Event-related synchronization in the gamma band. Brain 121, 2301–2315 10.1093/brain/121.12.2301 9874481

[B51] Crone N. E. Miglioretti D. L. Gordon B. Sieracki J. M. Wilson M. T. Uematsu S. Lesser R. P. (1998b). Functional mapping of human sensorimotor cortex with electrocorticographic spectral analysis—I. Alpha and beta event-related desynchronization. Brain 121, 2271–2299 10.1093/brain/121.12.2271 9874480

[B52] Daffertshofer A. Peper C. E. Beek P. J. (2005). Stabilization of bimanual coordination due to active interhemispheric inhibition: a dynamical account. Biol. Cybern. 92, 101–109 10.1007/s00422-004-0539-6 15685391

[B53] Daffertshofer A. van den Berg C. Beek P. J. (1999). A dynamical model for mirror movements. Physica D. 132, 243–266

[B54] Darvas F. Miller K. J. Rao R. P. N. Ojemann J. G. (2009). Nonlinear phase-phase cross-frequency coupling mediates communication between distant sites in human neocortex. J. Neurosci. 29, 426–435 10.1523/JNEUROSCI.3688-08.2009 19144842 PMC2745189

[B55] de Oliveira S. C. Gribova A. Donchin O. Bergman H. Vaadia E. (2001). Neural interactions between motor cortical hemispheres during bimanual and unimanual arm movements. Eur. J. Neurosci. 14, 1881–1896 10.1046/j.0953-816x.2001.01801.x 11860483

[B56] Defebvre L. Bourriez J. L. Derambure P. Duhamel A. Guieu J. D. Destee A. (1998). Influence of chronic administration of L-DOPA on event-related desynchronization of mu rhythm preceding voluntary movement in Parkinson's disease. Electroencephalogr. Clin. Neurophysiol. 109, 161–167 9741807 10.1016/s0924-980x(97)00085-4

[B57] Denker M. Roux S. Timme M. Riehle A. Grun S. (2007). Phase synchronization between LFP and spiking activity in motor cortex during movement preparation. Neurocomputing 70, 2096–2101

[B58] Dennis M. (1976). Impaired sensory and motor differentiation with corpus-callosum agenesis—lack of callosal inhibition during ontogeny. Neuropsychologia 14, 455–469 995239 10.1016/0028-3932(76)90074-9

[B59] Devos D. Labyt E. Cassim F. Bourriez J. L. Reyns N. Touzet G. Blond S. Guieu J. D. Derambure P. Destee A. Defebvre L. (2003a). Subthalamic stimulation influences postmovement cortical somatosensory processing in Parkinson's disease. Eur. J. Neurosci. 18, 1884–1888 10.1046/j.1460-9568.2003.02925.x 14622221

[B60] Devos D. Labyt E. Derambure P. Bourriez J. L. Cassim F. Guieu J. D. Destee A. Defebvre L. (2003b). Effect of L-Dopa on the pattern of movement-related (de)synchronisation in advanced Parkinson's disease. Clin. Neurophysiol. 33, 203–212 14672820 10.1016/j.neucli.2003.10.001

[B61] Donoghue J. P. Sanes J. N. Hatsopoulos N. G. Gaal G. (1998). Neural discharge and local field potential oscillations in primate motor cortex during voluntary movements. J. Neurophysiol. 79, 159–173 9425187 10.1152/jn.1998.79.1.159

[B62] Doyle L. M. F. Yarrow K. Brown P. (2005). Lateralization of event-related beta desynchronization in the EEG during pre-cued reaction time tasks. Clin. Neurophysiol. 116, 1879–1888 10.1016/j.clinph.2005.03.017 15979401

[B63] Duque J. Mazzocchio R. Dambrosia J. Murase N. Olivier E. Cohen L. G. (2005). Kinematically specific interhemispheric inhibition operating in the process of generation of a voluntary movement. Cereb. Cortex 15, 588–593 10.1093/cercor/bhh160 15342437

[B64] Eckhorn R. Bauer R. Jordan W. Brosch M. Kruse W. Munk M. Reitboeck H. J. (1988). Coherent oscillations—a mechanism of feature linking in the visual-cortex—multiple electrode and correlation analyses in the cat. Biol. Cybern. 60, 121–130 3228555 10.1007/BF00202899

[B65] Engel A. K. Fries P. (2010). Beta-band oscillations—signalling the status quo? Curr. Opin. Neurobiol. 20, 156–165 10.1016/j.conb.2010.02.015 20359884

[B66] Engel A. K. Konig P. Singer W. (1991). Direct physiological evidence for scene segmentation by temporal coding. Proc. Natl. Acad. Sci. U.S.A. 88, 9136–9140 1924376 10.1073/pnas.88.20.9136 PMC52667

[B67] Erbil N. Ungan P. (2007). Changes in the alpha and beta amplitudes of the central EEG during the onset, continuation, and offset of long-duration repetitive hand movements. Brain Res. 1169, 44–56 10.1016/j.brainres.2007.07.014 17689502

[B68] Farmer S. F. (1998). Rhythmicity, synchronization and binding in human and primate motor systems. J. Physiol. (Lond.) 509, 3–14 10.1111/j.1469-7793.1998.003bo.x 9547376 PMC2230956

[B69] Farmer S. F. Bremner F. D. Halliday D. M. Rosenberg J. R. Stephens J. A. (1993). The frequency content of common synaptic inputs to mononeurones studied during voluntary isometric contraction in man. J. Physiol. (Lond.) 470, 127–155 8308721 10.1113/jphysiol.1993.sp019851 PMC1143910

[B70] Farmer S. F. Harrison L. M. Mayston M. J. Parekh A. James L. M. Stephens J. A. (2004). Abnormal cortex-muscle interactions in subjects with X-linked Kallmann's syndrome and mirror movements. Brain 127, 385–397 10.1093/brain/awh047 14662517

[B71] Freeman W. J. (1975). Mass Action in the Nervous System. New York, NY: Academic Press

[B72] Freeman W. J. (1978). Spatial properties of an EEG event in olfactory-bulb and cortex. Electroencephalogr. Clin. Neurophysiol. 44, 586–605 77765 10.1016/0013-4694(78)90126-8

[B73] Freund H. J. (1983). Motor unit and muscle-activity in voluntary motor control. Physiol. Rev. 63, 387–436 6340133 10.1152/physrev.1983.63.2.387

[B74] Freyer F. Reinacher M. Nolte G. Dinse H. R. Ritter P. (2012). Repetitive tactile stimulation changes resting-state functional connectivity-implications for treatment of sensorimotor decline. Front. Hum. Neurosci. 6:144 10.3389/fnhum.2012.00144 22654748 PMC3358755

[B75] Fries P. Roelfsema P. R. Engel A. K. Konig P. Singer W. (1997). Synchronization of oscillatory responses in visual cortex correlates with perception in interocular rivalry. Proc. Natl. Acad. Sci. U.S.A. 94, 12699–12704 9356513 10.1073/pnas.94.23.12699 PMC25091

[B76] Fritsch G. Hitzig E. (1870). Über die elektrische erregbarkeit des grosshirns. Arch. Anat. Physiol. Wiss. Med. 37, 300–332 10.1016/j.yebeh.2009.03.002 19269348

[B77] Gastaut H. (1952). Etude electrocorticographique de la reactivite des rythmes rolandiques. Rev. Neurol. (Paris) 87, 176–182 13014777

[B78] Georgopoulos A. P. Schwartz A. B. Kettner R. E. (1986). Neuronal population coding of movement direction. Science 233, 1416–1419 10.1126/science.3749885 3749885

[B79] Gerloff C. Braun C. Staudt M. Hegner Y. L. Dichgans J. Krageloh-Mann I. (2006). Coherent corticomuscular oscillations originate from primary motor cortex: evidence from patients with early brain lesions. Hum. Brain Mapp. 27, 789–798 10.1002/hbm.20220 16475178 PMC6871432

[B80] Gerloff C. Richard J. Hadley J. Schulman A. E. Honda M. Hallett M. (1998). Functional coupling and regional activation of human cortical motor areas during simple, internally paced and externally paced finger movements. Brain 121, 1513–1531 10.1093/brain/121.8.1513 9712013

[B81] Gilbertson T. Lalo E. Doyle L. Di Lazzaro V. Cioni B. Brown P. (2005). Existing motor state is favored at the expense of new movement during 13–35 Hz oscillatory synchrony in the human corticospinal system. J. Neurosci. 25, 7771–7779 10.1523/JNEUROSCI.1762-05.2005 16120778 PMC6725263

[B82] Goldberg J. A. Boraud T. Maraton S. Haber S. N. Vaadia E. Bergman H. (2002). Enhanced synchrony among primary motor cortex neurons in the 1-methyl-4-phenyl-1 2 3, 6-tetrahydropyridine primate model of Parkinson's disease. J. Neurosci. 22, 4639–4653 12040070 10.1523/JNEUROSCI.22-11-04639.2002 PMC6758785

[B83] Golledge H. D. R. Panzeri S. Zheng F. S. Pola G. Scannell J. W. Giannikopoulos D. V. Mason R. J. Tovee M. J. Young M. P. (2003). Correlations, feature-binding and population coding in primary visual cortex. Neuroreport 14, 1045–1050 10.1097/01.wnr.0000073681.00308.9c 12802200

[B84] Gomez-Herrero G. Atienza M. Egiazarian K. Cantero J. L. (2008). Measuring directional coupling between EEG sources. Neuroimage 43, 497–508 10.1016/j.neuroimage.2008.07.032 18707006

[B85] Grammont F. Riehle A. (2003). Spike synchronization and firing rate in a population of motor cortical neurons in relation to movement direction and reaction time. Biol. Cybern. 88,360–373 10.1007/s00422-002-0385-3 12750898

[B86] Gray C. M. Konig P. Engel A. K. Singer W. (1989). Oscillatory responses in cat visual-cortex exhibit inter-columnar synchronization which reflects global stimulus properties. Nature 338, 334–337 10.1038/338334a0 2922061

[B87] Gross J. Pollok B. Dirks M. Timmermann L. Butz M. Schnitzler A. (2005). Task-dependent oscillations during unimanual and bimanual movements in the human primary motor cortex and SMA studied with magnetoencephalography. Neuroimage 26, 91–98 10.1016/j.neuroimage.2005.01.025 15862209

[B88] Gross J. Tass P. A. Salenius S. Hari R. Freund H. J. Schnitzler A. (2000). Cortico-muscular synchronization during isometric muscle contraction in humans as revealed by magnetoencephalography. J. Physiol. (Lond.) 527, 623–631 10.1111/j.1469-7793.2000.00623.x 10990546 PMC2270094

[B89] Gross J. Timmermann J. Kujala J. Dirks M. Schmitz F. Salmelin R. Schnitzler A. (2002). The neural basis of intermittent motor control in humans. Proc. Natl. Acad. Sci. U.S.A. 99, 2299–2302 10.1073/pnas.032682099 11854526 PMC122359

[B90] Halliday D. M. Conway B. A. Farmer S. F. Rosenberg J. R. (1998). Using electroencephalography to study functional coupling between cortical activity and electromyograms during voluntary contractions in humans. Neurosci. Lett. 241, 5–8 10.1016/S0304-3940(97)00964-6 9502202

[B91] Hammond C. Bergman H. Brown P. (2007). Pathological synchronization in Parkinson's disease: networks, models and treatments. Trends Neurosci. 30, 357–364 10.1016/j.tins.2007.05.004 17532060

[B92] Hatsopoulos N. G. Ojakangas C. L. Paninski L. Donoghue J. P. (1998). Information about movement direction obtained from synchronous activity of motor cortical neurons. Proc. Natl. Acad. Sci. U.S.A. 95, 15706–15711 10.1073/pnas.95.26.15706 9861034 PMC28108

[B93] Hauptmann C. Roulet J. C. Niederhauser J. J. Doll W. Kirlangic M. E. Lysyansky B. Krachkovskyi V. Bhatti M. A. Barnikol U. B. Sasse L. Buhrle C. P. Speckmann E. J. Gotz M. Sturm V. Freund H. J. Schnell U. Tass P. A. (2009). External trial deep brain stimulation device for the application of desynchronizing stimulation techniques. J. Neural Eng. 6, 066003 10.1088/1741-2560/6/6/066003 19837998

[B94] Hellwig B. Haussler S. Schelter B. Lauk M. Guschlbauer B. Timmer J. Lucking C. H. (2001). Tremor-correlated cortical activity in essential tremor. Lancet 357, 519–523 10.1016/S0140-6736(00)04044-7 11229671

[B95] Hogan K. Fitzpatrick J. (1988). The cerebral origin of the alpha-rhythm. Electroencephalogr. Clin. Neurophysiol. 69, 79–81 2448124 10.1016/0013-4694(88)90037-5

[B96] Hommel B. (2004). Event files: feature binding in and across perception and action. Trends Cogn. Sci. 8, 494–500 10.1016/j.tics.2004.08.007 15491903

[B97] Houweling S. Beek P. J. Daffertshofer A. (2010a). Spectral changes of interhemispheric crosstalk during movement instabilities. Cereb. Cortex 20, 2605–2613 10.1093/cercor/bhq008 20176689

[B98] Houweling S. van Dijk B. W. Beek P. J. Daffertshofer A. (2010b). Cortico-spinal synchronization reflects changes in performance when learning a complex bimanual task. Neuroimage 49, 3269–3275 10.1016/j.neuroimage.2009.11.017 19922805

[B99] Houweling S. Daffertshofer A. van Dijk B. W. Beek P. J. (2008). Neural changes induced by learning a challenging perceptual-motor task. Neuroimage 41, 1395–1407 10.1016/j.neuroimage.2008.03.023 18485745

[B100] Hughes S. W. Crunelli V. (2005). Thalamic mechanisms of EEG alpha rhythms and their pathological implications. Neuroscientist 11, 357–372 10.1177/1073858405277450 16061522

[B101] Hummel F. Kirsammer R. Gerloff C. (2003). Ipsilateral cortical activation during finger sequences of increasing complexity: representation of movement difficulty or memory load? Clin. Neurophysiol. 114, 605–613 10.1016/S1388-2457(02)00417-0 12686269

[B102] Huo X. L. Wang Y. Y. Kotecha R. Kirtman E. G. Fujiwara H. Hemasilpin N. Degrauw T. Rose D. F. Xiang J. (2011). High gamma oscillations of sensorimotor cortex during unilateral movement in the developing brain: a MEG study. Brain Topogr. 23, 375–384 10.1007/s10548-010-0151-0 20577795

[B103] Jackson A. Gee V. J. Baker S. N. Lemon R. N. (2003). Synchrony between neurons with similar muscle fields in monkey motor cortex. Neuron 38, 115–125 10.1016/S0896-6273(03)00162-4 12691669

[B104] Jasper H. Penfield W. (1949). Electrocorticograms in man: effect of voluntary movement upon the electrical activity of the precentral gyrus. Arch. Psychiat. Z. Neurol. 183, 163–174

[B105] Jasper H. H. Andrews H. L. (1938). Electro-encephalography. III. Normal differentiation of occipital and precentral regions in man. Arch. Neurol. Psychiatry 39, 96–115

[B106] Jenkinson N. Brown P. (2011). New insights into the relationship between dopamine, beta oscillations and motor function. Trends Neurosci. 34, 611–618 10.1016/j.tins.2011.09.003 22018805

[B107] Jin S. H. Lin P. Hallett M. (2010). Linear and nonlinear information flow based on time-delayed mutual information method and its application to corticomuscular interaction. Clin. Neurophysiol. 121, 392–401 10.1016/j.clinph.2009.09.033 20044309 PMC2822094

[B108] Johnson A. N. Wheaton L. A. Shinohara M. (2011). Attenuation of corticomuscular coherence with additional motor or non-motor task. Clin. Neurophysiol. 122, 356–363 10.1016/j.clinph.2010.06.021 20638330

[B109] Keller A. (1993). Intrinsic synaptic organization of the motor cortex. Cereb. Cortex 3, 430–441 8260811 10.1093/cercor/3.5.430

[B110] Kilner J. M. Alonso-Alonso M. Fisher R. Lemon R. N. (2002). Modulation of synchrony between single motor units during precision grip tasks in humans. J. Physiol. (Lond.) 541, 937–948 10.1113/jphysiol.2001.013305 12068052 PMC2290366

[B111] Kilner J. M. Baker S. N. Salenius S. Hari R. Lemon R. N. (2000). Human cortical muscle coherence is directly related to specific motor parameters. J. Neurosci. 20, 8838–8845 11102492 10.1523/JNEUROSCI.20-23-08838.2000 PMC6773054

[B112] Kilner J. M. Baker S. N. Salenius S. Jousmaki V. Hari R. Lemon R. N. (1999). Task-dependent modulation of 15–30 Hz coherence between rectified EMGs from human hand and forearm muscles. J. Physiol. (Lond.) 516, 559–570 10.1111/j.1469-7793.1999.0559v.x 10087353 PMC2269269

[B113] Kim M. S. Masakado Y. Tomita Y. Chino N. Pae Y. S. Lee K. (2001). Synchronization of single motor units during voluntary contractions in the upper and lower extremities. Clin. Neurophysiol. 112, 1243–1249 11516736 10.1016/s1388-2457(01)00549-1

[B114] Klimesch W. Sauseng P. Hanslmayr S. (2007). EEG alpha oscillations: the inhibition-timing hypothesis. Brain Res. Rev. 53, 63–88 10.1016/j.brainresrev.2006.06.003 16887192

[B115] Konig P. Engel A. K. Singer W. (1995). Relation between oscillatory activity and long-range synchronization in cat visual-cortex. Proc. Natl. Acad. Sci. U.S.A. 92, 290–294 7816836 10.1073/pnas.92.1.290 PMC42864

[B116] Kreiter A. K. Singer W. (1996). Stimulus-dependent synchronization of neuronal responses in the visual cortex of the awake macaque monkey. J. Neurosci. 16, 2381–2396 8601818 10.1523/JNEUROSCI.16-07-02381.1996 PMC6578521

[B117] Kristeva R. Patino L. Omlor W. (2007). Beta-range cortical motor spectral power and corticomuscular coherence as a mechanism for effective corticospinal interaction during steady-state motor output. Neuroimage 36, 785–792 10.1016/j.neuroimage.2007.03.025 17493837

[B118] Kristeva-Feige R. Fritsch C. Timmer J. Lucking C. H. (2002). Effects of attention and precision of exerted force on beta range EEG-EMG synchronization during a maintained motor contraction task. Clin. Neurophysiol. 113, 124–131 11801434 10.1016/s1388-2457(01)00722-2

[B119] Kuhn A. A. Kempf F. Brucke C. Doyle L. G. Martinez-Torres I. Pogosyan A. Trottenberg T. Kupsch A. Schneider G. H. Hariz M. I. Vandenberghe W. Nuttin B. Brown P. (2008). High-frequency stimulation of the subthalamic nucleus suppresses oscillatory beta activity in patients with Parkinson's disease in parallel with improvement in motor performance. J. Neurosci. 28, 6165–6173 10.1523/JNEUROSCI.0282-08.2008 18550758 PMC6670522

[B120] Kuhn A. A. Kupsch A. Schneider G. H. Brown P. (2006). Reduction in subthalamic 8–35 Hz oscillatory activity correlates with clinical improvement in Parkinson's disease. Eur. J. Neurosci. 23, 1956–1960 10.1111/j.1460-9568.2006.04717.x 16623853

[B121] Kuhn A. A. Tsui A. Aziz T. Ray N. Brucke C. Kupsch A. Schneider G. H. Brown P. (2009). Pathological synchronisation in the subthalamic nucleus of patients with Parkinson's disease relates to both bradykinesia and rigidity. Exp. Neurol. 215, 380–387 10.1016/j.expneurol.2008.11.008 19070616

[B122] Lachaux J. P. Rodriguez E. Martinerie J. Varela F. J. (1999). Measuring phase synchrony in brain signals. Hum. Brain Mapp. 8, 194–208 10.1002/(SICI)1097-0193(1999)8:4<194::AID-HBM4>3.0.CO;2-C 10619414 PMC6873296

[B123] Latham P. E. Nirenberg S. (2005). Synergy, redundancy, and independence in population codes, revisited. J. Neurosci. 25, 5195–5206 10.1523/JNEUROSCI.5319-04.2005 15917459 PMC6724819

[B124] Lemon R. N. Vanderburg J. (1979). Short-latency peripheral inputs to thalamic neurons projecting to the motor cortex in the monkey. Exp. Brain Res. 36, 445–462 113234 10.1007/BF00238515

[B125] Leocani L. Cohen L. G. Wassermann E. M. Ikoma K. Hallett M. (2000). Human corticospinal excitability evaluated with transcranial magnetic stimulation during different reaction time paradigms. Brain 123, 1161–1173 10.1093/brain/123.6.1161 10825355

[B126] Leuthardt E. C. Schalk G. Wolpaw J. R. Ojemann J. G. Moran D. W. (2004). A brain-computer interface using electrocorticographic signals in humans. J. Neural Eng. 1, 63–71 10.1088/1741-2560/1/2/001 15876624

[B127] Lopes Da Silva F. H. (1991). Neural mechanisms underlying brain waves—from neural membranes to networks. Electroencephalogr. Clin. Neurophysiol. 79, 81–93 1713832 10.1016/0013-4694(91)90044-5

[B128] Lopes Da Silva F. H. Hoeks A. Smits H. Zetterberg L. H. (1974). Model of brain rhythmic activity—alpha-rhythm of thalamus. Kybernetik 15, 27–37 4853232 10.1007/BF00270757

[B129] Lorenz D. Deuschl G. (2007). Update on pathogenesis and treatment of essential tremor. Curr. Opin. Neurol. 20, 447–452 10.1097/WCO.0b013e3281e66942 17620881

[B130] Lumer E. D. Edelman G. M. Tononi G. (1997a). Neural dynamics in a model of the thalamocortical system. 1. Layers, loops and the emergence of fast synchronous rhythms. Cereb. Cortex 7, 207–227 10.1093/cercor/7.3.207 9143442

[B131] Lumer E. D. Edelman G. M. Tononi G. (1997b). Neural dynamics in a model of the thalamocortical system. 2. The role of neural synchrony tested through perturbations of spike timing. Cereb. Cortex 7, 228–236 10.1093/cercor/7.3.228 9143443

[B132] Lytton W. W. Sejnowski T. J. (1991). Simulations of cortical pyramidal neurons synchronized by inhibitory interneurons. J. Neurophysiol. 66, 1059–1079 1661324 10.1152/jn.1991.66.3.1059

[B133] Magnani G. Cursi M. Leocani L. Volonte M. A. Comi G. (2002). Acute effects of L-dopa on event-related desynchronization in Parkinson's disease. Neurol. Sci. 23, 91–97 10.1007/s100720200033 12391492

[B134] Manganotti P. Gerloff C. Toro C. Katsuta H. Sadato N. Zhuang P. Leocani L. Hallett M. (1998). Task-related coherence and task-related spectral power changes during sequential finger movements. Electroencephalogr. Clin. Neurophysiol. 109, 50–62 11003064 10.1016/s0924-980x(97)00074-x

[B135] Mardia K. V. (1972). Statistics of Directional Data. London/New York: Academic Press

[B136] Marsden J. F. Farmer S. F. Halliday D. M. Rosenberg J. R. Brown P. (1999). The unilateral and bilateral control of motor unit pairs in the first dorsal interosseous and paraspinal muscles in man. J. Physiol. (Lond.) 521, 553–564 10.1111/j.1469-7793.1999.00553.x 10581324 PMC2269660

[B137] Marsden J. F. Werhahn K. J. Ashby P. Rothwell J. Noachtar S. Brown P. (2000). Organization of cortical activities related to movement in humans. J. Neurosci. 20, 2307–2314 10704506 10.1523/JNEUROSCI.20-06-02307.2000 PMC6772500

[B138] Marzetti L. Del Gratta C. Nolte G. (2008). Understanding brain connectivity from EEG data by identifying systems composed of interacting sources. Neuroimage 42, 87–98 10.1016/j.neuroimage.2008.04.250 18539485

[B139] Meinecke F. C. Ziehe A. Kurths J. Muller K. R. (2005). Measuring phase synchronization of superimposed signals. Phys. Rev. Lett. 94, 084102 10.1103/PhysRevLett.94.084102 15783894

[B140] Miller K. J. Leuthardt E. C. Schalk G. Rao R. P. N. Anderson N. R. Moran D. W. Miller J. W. Ojemann J. G. (2007). Spectral changes in cortical surface potentials during motor movement. J. Neurosci. 27, 2424–2432 10.1523/JNEUROSCI.3886-06.2007 17329441 PMC6673496

[B141] Mima T. Matsuoka T. Hallett M. (2000a). Functional coupling of human right and left cortical motor areas demonstrated with partial coherence analysis. Neurosci. Lett. 287, 93–96 10.1186/1471-2202-10-22 10854720

[B142] Mima T. Steger J. Schulman A. E. Gerloff C. Hallett M. (2000b). Electroencephalographic measurement of motor cortex control of muscle activity in humans. Clin. Neurophysiol. 111, 326–337 10680569 10.1016/s1388-2457(99)00229-1

[B143] Mima T. Matsuoka T. Hallett M. (2001). Information flow from the sensorimotor cortex to muscle in humans. Clin. Neurophysiol. 112, 122–126 11137669 10.1016/s1388-2457(00)00515-0

[B144] Mima T. Simpkins N. Oluwatimilehin T. Hallett M. (1999). Force level modulates human cortical oscillatory activities. Neurosci. Lett. 275, 77–80 10.1016/S0304-3940(99)00734-X 10568503

[B145] Murthy V. N. Fetz E. E. (1992). Coherent 25-Hz to 35-Hz oscillations in the sensorimotor cortex of awake behaving monkeys. Proc. Natl. Acad. Sci. U.S.A. 89, 5670–5674 1608977 10.1073/pnas.89.12.5670 PMC49354

[B146] Murthy V. N. Fetz E. E. (1996a). Oscillatory activity in sensorimotor cortex of awake monkeys: synchronization of local field potentials and relation to behavior. J. Neurophysiol. 76, 3949–3967 8985892 10.1152/jn.1996.76.6.3949

[B147] Murthy V. N. Fetz E. E. (1996b). Synchronization of neurons during local field potential oscillations in sensorimotor cortex of awake monkeys. J. Neurophysiol. 76, 3968–3982 8985893 10.1152/jn.1996.76.6.3968

[B148] Muskulus M. Houweling S. Verduyn-Lunel S. Daffertshofer A. (2009). Functional similarities and distance properties. J. Neurosci. Methods 183, 31–41 10.1016/j.jneumeth.2009.06.035 19589355

[B149] Muthukumaraswamy S. D. (2010). Functional properties of human primary motor cortex gamma oscillations. J. Neurophysiol. 104, 2873–2885 10.1152/jn.00607.2010 20884762

[B150] Muthuraman M. Govindan R. B. Deuschl G. Heute U. Raethjen J. (2008). Differentiating phase shift and delay in narrow band coherent signals. Clin. Neurophysiol. 119, 1062–1070 10.1016/j.clinph.2008.01.003 18308625

[B151] Myers L. J. Lowery M. O'Malley M. Vaughan C. L. Heneghan C. Gibson A. S. C. Harley Y. X. R. Sreenivasan R. (2003). Rectification and non-linear pre-processing of EMG signals for cortico-muscular analysis. J. Neurosci. Methods 124, 157–165 10.1016/S0165-0270(03)00004-9 12706845

[B152] Negro F. Farina D. (2011). Decorrelation of cortical inputs and motoneuron output. J. Neurophysiol. 106, 2688–2697 10.1152/jn.00336.2011 21795617

[B153] Neuper C. Pfurtscheller G. (2001). Event-related dynamics of cortical rhythms: frequency-specific features and functional correlates. Int. J. Psychophysiol. 43, 41–58 10.1016/S0167-8760(01)00178-7 11742684

[B154] Nirenberg S. Carcieri S. M. Jacobs A. L. Latham P. E. (2001). Retinal ganglion cells act largely as independent encoders. Nature 411, 698–701 10.1038/35079612 11395773

[B155] Nolte G. Bai O. Wheaton L. Mari Z. Vorbach S. Hallett M. (2004). Identifying true brain interaction from EEG data using the imaginary part of coherency. Clin. Neurophysiol. 115, 2292–2307 10.1016/j.clinph.2004.04.029 15351371

[B156] Nolte G. Ziehe A. Nikulin V. V. Schlogl A. Kramer N. Brismar T. Muller K. R. (2008). Robustly estimating the flow direction of information in complex physical systems. Phys. Rev. Lett. 100, 234101 10.1103/PhysRevLett.100.234101 18643502

[B157] Ohara S. Mima T. Baba K. Ikeda A. Kunieda T. Matsumoto R. Yamamoto J. Matsuhashi M. Nagamine T. Hirasawa K. Hori T. Mihara T. Hashimoto N. Salenius S. Shibasaki H. (2001). Increased synchronization of cortical oscillatory activities between human supplementary motor and primary sensorimotor areas during voluntary movements. J. Neurosci. 21, 9377–9386 11717371 10.1523/JNEUROSCI.21-23-09377.2001 PMC6763917

[B158] Omlor W. Patino L. Hepp-Reymond M. C. Kristeva R. (2007). Gamma-range corticomuscular coherence during dynamic force output. Neuroimage 34, 1191–1198 10.1016/j.neuroimage.2006.10.018 17182258

[B159] Omlor W. Patino L. Mendez-Balbuena I. Schulte-Monting J. Kristeva R. (2011). Corticospinal beta-range coherence is highly dependent on the pre-stationary motor state. J. Neurosci. 31, 8037–8045 10.1523/JNEUROSCI.4153-10.2011 21632925 PMC6622845

[B160] Oram M. W. Hatsopoulos N. G. Richmond B. J. Donoghue J. P. (2001). Excess synchrony in motor cortical neurons provides redundant direction information with that from coarse temporal measures. J. Neurophysiol. 86, 1700–1716 11600633 10.1152/jn.2001.86.4.1700

[B161] Penfield W. Boldrey E. (1937). Somatic motor and sensory representation in the cerebral cortex of man as studied by electrical stimulation. Brain 60, 389–443

[B162] Pereda E. Quiroga R. Q. Bhattacharya J. (2005). Nonlinear multivariate analysis of neurophysiological signals. Prog. Neurobiol. 77, 1–37 10.1016/j.pneurobio.2005.10.003 16289760

[B163] Pfurtscheller G. Graimann B. Huggins J. E. Levine S. P. Schuh L. A. (2003). Spatiotemporal patterns of beta desynchronization and gamma synchronization in corticographic data during self-paced movement. Clin. Neurophysiol. 114, 1226–1236 10.1016/S1388-2457(03)00067-1 12842719

[B164] Pfurtscheller G. Lopes Da Silva F. H. (1999). Event-related EEG/MEG synchronization and desynchronization: basic principles. Clin. Neurophysiol. 110, 1842–1857 10576479 10.1016/s1388-2457(99)00141-8

[B165] Pfurtscheller G. Neuper C. (1992). Simultaneous EEG 10-Hz desynchronization and 40-Hz synchronization during finger movements. Neuroreport 3, 1057–1060 1493217 10.1097/00001756-199212000-00006

[B166] Pfurtscheller G. Neuper C. Kalcher J. (1993). 40-Hz oscillations during motor behavior in man. Neurosci. Lett. 164, 179–182 10.1016/0304-3940(93)90886-P 8152598

[B167] Pfurtscheller G. Stancak A. Neuper C. (1996). Post-movement beta synchronization. A correlate of an idling motor area? Electroencephalogr. Clin. Neurophysiol. 98, 281–293 8641150 10.1016/0013-4694(95)00258-8

[B168] Pfurtscheller G. Zalaudek K. Neuper C. (1998). Event-related beta synchronization after wrist, finger and thumb movement. Electroencephalogr. Clin. Neurophysiol. 109, 154–160 9741806 10.1016/s0924-980x(97)00070-2

[B169] Pogosyan A. Gaynor L. D. Eusebio A. Brown P. (2009). Boosting cortical activity at beta-band frequencies slows movement in humans. Curr. Biol. 19, 1637–1641 10.1016/j.cub.2009.07.074 19800236 PMC2791174

[B170] Pohja M. Salenius S. Hari R. (2002). Cortico-muscular coupling in a human subject with mirror movements—a magnetoencephalographic study. Neurosci. Lett. 327, 185–188 10.1016/S0304-3940(02)00426-3 12113908

[B171] Pollok B. Butz M. Gross J. Schnitzler A. (2007). Intercerebellar coupling contributes to bimanual coordination. J. Cogn. Neurosci. 19, 704–719 10.1162/jocn.2007.19.4.704 17381260

[B172] Pollok B. Gross J. Muller K. Aschersleben G. Schnitzler A. (2005). The cerebral oscillatory network associated with auditorily paced finger movements. Neuroimage 24, 646–655 10.1016/j.neuroimage.2004.10.009 15652300

[B173] Priori A. Foffani G. Pesenti A. Tamma F. Bianchi A. M. Pellegrini M. Locatelli M. Moxon K. A. Villani R. M. (2004). Rhythm-specific pharmacological modulation of subthalamic activity in Parkinson's disease. Exp. Neurol. 189, 369–379 10.1016/j.expneurol.2004.06.001 15380487

[B174] Raethjen J. Deuschl G. (2012). The oscillating central network of essential tremor. Clin. Neurophysiol. 123, 61–64 10.1016/j.clinph.2011.09.024 22055842

[B175] Raethjen J. Govindan R. B. Kopper F. Muthuraman M. Deuschl G. (2007). Cortical involvement in the generation of essential tremor. J. Neurophysiol. 97, 3219–3228 10.1152/jn.00477.2006 17344375

[B176] Ray N. J. Jenkinson N. Wang S. Holland P. Brittain J. S. Joint C. Stein J. F. Aziz T. (2008). Local field potential beta activity in the subthalamic nucleus of patients with Parkinson's disease is associated with improvements in bradykinesia after dopamine and deep brain stimulation. Exp. Neurol. 213, 108–113 10.1016/j.expneurol.2008.05.008 18619592

[B177] Rickert J. de Oliveira S. C. Vaadia E. Aertsen A. Rotter S. Mehring C. (2005). Encoding of movement direction in different frequency ranges of motor cortical local field potentials. J. Neurosci. 25, 8815–8824 10.1523/JNEUROSCI.0816-05.2005 16192371 PMC6725584

[B178] Riddle C. N. Baker M. R. Baker S. N. (2004). The effect of carbamazepine on human corticomuscular coherence. Neuroimage 22, 333–340 10.1016/j.neuroimage.2003.12.040 15110023

[B179] Riddle C. N. Baker S. N. (2005). Manipulation of peripheral neural feedback loops alters human corticomuscular coherence. J. Physiol. (Lond.) 566, 625–639 10.1113/jphysiol.2005.089607 15919711 PMC1464768

[B180] Riddle C. N. Baker S. N. (2006). Digit displacement, not object compliance, underlies task dependent modulations in human corticomuscular coherence. Neuroimage 33, 618–627 10.1016/j.neuroimage.2006.07.027 16963283

[B181] Riehle A. Grun S. Diesmann M. Aertsen A. (1997). Spike synchronization and rate modulation differentially involved in motor cortical function. Science 278, 1950–1953 10.1126/science.278.5345.1950 9395398

[B182] Ritter P. Moosmann M. Villringer A. (2009). Rolandic alpha and beta EEG rhythms' strengths are inversely related to fMRI-BOLD signal in primary somatosensory and motor cortex. Hum. Brain Mapp. 30, 1168–1187 10.1002/hbm.20585 18465747 PMC6870597

[B183] Roelfsema P. R. Engel A. K. Konig P. Singer W. (1996). The role of neuronal synchronization in response selection: a biologically plausible theory of structured representations in the visual cortex. J. Cogn. Neurosci. 8, 603–625 10.1162/jocn.1996.8.6.603 23961987

[B184] Roelfsema P. R. Lamme V. A. F. Spekreijse H. (2004). Synchrony and covariation of firing rates in the primary visual cortex during contour grouping. Nat. Neurosci. 7, 982–991 10.1038/nn1304 15322549

[B185] Safri N. M. Murayama N. Hayashida Y. Igasaki T. (2007). Effects of concurrent visual tasks on cortico-muscular synchronization in humans. Brain Res. 1155, 81–92 10.1016/j.brainres.2007.04.052 17512919

[B186] Salenius S. Avikainen S. Kaakkola S. Hari R. Brown P. (2002). Defective cortical drive to muscle in Parkinson's disease and its improvement with levodopa. Brain 125, 491–500 10.1093/brain/awf042 11872607

[B187] Salenius S. Hari R. (2003). Synchronous cortical oscillatory activity during motor action. Curr. Opin. Neurobiol. 13, 678–684 10.1016/j.conb.2003.10.008 14662368

[B188] Salenius S. Portin K. Kajola M. Salmelin R. Hari R. (1997). Cortical control of human motoneuron firing during isometric contraction. J. Neurophysiol. 77, 3401–3405 9212286 10.1152/jn.1997.77.6.3401

[B189] Salmelin R. Hamalainen M. Kajola M. Hari R. (1995). Functional segregation of movement-related rhythmic activity in the human brain. Neuroimage 2, 237–243 10.1006/nimg.1995.1031 9343608

[B190] Salmelin R. Hari R. (1994). Spatiotemporal characteristics of sensorimotor neuromagnetic rhythms related to thumb movement. Neuroscience 60, 537–550 10.1016/0306-4522(94)90263-1 8072694

[B191] Sanes J. N. Donoghue J. P. (1993). Oscillations in local-field potentials of the primate motor cortex during voluntary movement. Proc. Natl. Acad. Sci. U.S.A. 90, 4470–4474 8506287 10.1073/pnas.90.10.4470 PMC46533

[B192] Schnitzler A. Munks C. Butz M. Timmermann L. Gross J. (2009). Synchronized brain network associated with essential tremor as revealed by magnetoencephalography. Mov. Disord. 24, 1629–1635 10.1002/mds.22633 19514010

[B193] Schnitzler A. Timmermann L. Gross J. (2006). Physiological and pathological oscillatory networks in the human motor system. J. Physiol. Paris 99, 3–7 10.1016/j.jphysparis.2005.06.010 16054347

[B194] Schoffelen J. M. Gross J. (2009). Source connectivity analysis with MEG and EEG. Hum. Brain Mapp. 30, 1857–1865 10.1002/hbm.20745 19235884 PMC6870611

[B195] Schoffelen J. M. Oostenveld R. Fries P. (2005). Neuronal coherence as a mechanism of effective corticospinal interaction. Science 308, 111–113 10.1126/science.1107027 15802603

[B196] Serrien D. J. (2008). The neural dynamics of timed motor tasks: evidence from a synchronization-continuation paradigm. Eur. J. Neurosci. 27, 1553–1560 10.1111/j.1460-9568.2008.06110.x 18336571

[B197] Serrien D. J. Brown P. (2002). The functional role of interhemispheric synchronization in the control of bimanual timing tasks. Exp. Brain Res. 147, 268–272 10.1007/s00221-002-1253-z 12410342

[B198] Serrien D. J. Brown P. (2003). The integration of cortical and behavioural dynamics during initial learning of a motor task. Eur. J. Neurosci. 17, 1098–1104 10.1046/j.1460-9568.2003.02534.x 12653986

[B199] Shadlen M. N. Movshon J. A. (1999). Synchrony unbound: a critical evaluation of the temporal binding hypothesis. Neuron 24, 67–77 10.1016/S0896-6273(00)80822-3 10677027

[B200] Shibasaki H. Nagae K. (1984). Mirror movement—application of movement-related cortical potentials. Ann. Neurol. 15, 299–302 10.1002/ana.410150317 6721452

[B201] Silberstein P. Pogosyan A. Kuhn A. A. Hotton G. Tisch S. Kupsch A. Dowsey-Limousin P. Hariz M. I. Brown P. (2005). Cortico-cortical coupling in Parkinson's disease and its modulation by therapy. Brain 128, 1277–1291 10.1093/brain/awh480 15774503

[B202] Singer W. (1994). The organization of sensory motor representations in the neocortex: a hypothesis based on temporal coding, in Attention and Performance XV: Conscious and Nonconscious Information Processing, eds Umiltà C. Moscovitch M. (Cambridge, MA: MIT Press), 77–107

[B203] Singer W. Gray C. M. (1995). Visual feature integration and the temporal correlation hypothesis. Annu. Rev. Neurosci. 18, 555–586 10.1146/annurev.ne.18.030195.003011 7605074

[B204] Soteropoulos D. S. Baker S. N. (2006). Cortico-cerebellar coherence during a precision grip task in the monkey. J. Neurophysiol. 95, 1194–1206 10.1152/jn.00935.2005 16424458

[B205] Spinks R. L. Kraskov A. Brochier T. Umilta M. A. Lemon R. N. (2008). Selectivity for grasp in local field potential and single neuron activity recorded simultaneously from M1 and F5 in the awake macaque monkey. J. Neurosci. 28, 10961–10971 10.1523/JNEUROSCI.1956-08.2008 18945904 PMC2637078

[B206] Srinivasan R. Winter W. R. Ding J. Nunez P. L. (2007). EEG and MEG coherence: measures of functional connectivity at distinct spatial scales of neocortical dynamics. J. Neurosci. Methods 166, 41–52 10.1016/j.jneumeth.2007.06.026 17698205 PMC2151962

[B207] Stam C. J. Nolte G. Daffertshofer A. (2007). Phase lag index: assessment of functional connectivity from multi channel EEG and MEG with diminished bias from common sources. Hum. Brain Mapp. 28, 1178–1193 10.1002/hbm.20346 17266107 PMC6871367

[B208] Stancak A. Feige B. Lucking C. H. Kristeva-Feige R. (2000). Oscillatory cortical activity and movement-related potentials in proximal and distal movements. Clin. Neurophysiol. 111, 636–650 10727915 10.1016/s1388-2457(99)00310-7

[B209] Stancak A. Pfurtscheller G. (1996). Mu-rhythm changes in brisk and slow self-paced finger movements. Neuroreport 7, 1161–1164 8817524 10.1097/00001756-199604260-00013

[B210] Stancak A. Riml A. Pfurtscheller G. (1997). The effects of external load on movement-related changes of the sensorimotor EEG rhythms. Electroencephalogr. Clin. Neurophysiol. 102, 495–504 9216482 10.1016/s0013-4694(96)96623-0

[B211] Stark E. Globerson A. Asher I. Abeles M. (2008). Correlations between groups of premotor neurons carry information about prehension. J. Neurosci. 28, 10618–10630 10.1523/JNEUROSCI.3418-08.2008 18923038 PMC6671346

[B212] Steriade M. Gloor P. Llinas R. R. Dasilva F. H. L. Mesulam M. M. (1990). Basic mechanisms of cerebral rhythmic activities. Electroencephalogr. Clin. Neurophysiol. 76, 481–508 1701118 10.1016/0013-4694(90)90001-z

[B213] Swann N. Tandon N. Canolty R. Ellmore T. M. McEvoy L. K. Dreyer S. DiSano M. Aron A. R. (2009). Intracranial EEG reveals a time- and frequency-specific role for the right inferior frontal gyrus and primary motor cortex in stopping initiated responses. J. Neurosci. 29, 12675–12685 10.1523/JNEUROSCI.3359-09.2009 19812342 PMC2801605

[B214] Szurhaj W. Bourriez J. L. Kahane P. Chauvel P. Mauguiere F. Derambure P. (2005). Intracerebral study of gamma rhythm reactivity in the sensorimotor cortex. Eur. J. Neurosci. 21, 1223–1235 10.1111/j.1460-9568.2005.03966.x 15813932

[B215] Tass P. Rosenblum M. G. Weule J. Kurths J. Pikovsky A. Volkmann J. Schnitzler A. Freund H. J. (1998). Detection of n:m phase locking from noisy data: application to magnetoencephalography. Phys. Rev. Lett. 81, 3291–3294

[B216] Tass P. A. (1999). Phase Resetting in Medicine and Biology—Stochastic Modelling and Data Analysis. Berlin: Springer

[B217] Tass P. A. (2002). Desynchronization of brain rhythms with soft phase-resetting techniques. Biol. Cybern. 87, 102–115 10.1007/s00422-002-0322-5 12181586

[B218] Tognoli E. Kelso J. A. S. (2009). Brain coordination dynamics: true and false faces of phase synchrony and metastability. Prog. Neurobiol. 87, 31–40 10.1016/j.pneurobio.2008.09.014 18938209 PMC3020160

[B219] Toma K. Mima T. Matsuoka T. Gerloff C. Ohnishi T. Koshy B. Andres F. Hallett M. (2002). Movement rate effect on activation and functional coupling of motor cortical areas. J. Neurophysiol. 88, 3377–3385 10.1152/jn.00281.2002 12466454

[B220] Tsujimoto T. Mima T. Shimazu H. Isomura Y. (2009). Directional organization of sensorimotor oscillatory activity related to the electromyogram in the monkey. Clin. Neurophysiol. 120, 1168–1173 10.1016/j.clinph.2009.02.177 19394270

[B221] Vaadia E. Ahissar E. Bergman H. Lavner Y. (1991). Correlated activity of neurons: a neural code for higher brain functions? in Neuronal Cooperativity, ed Krüger J. (Berlin/Heidelberg/New York: Springer Verlag), 249–279

[B222] van Wijk B. C. M. Beek P. J. Daffertshofer A. (2012). Differential modulations of ipsilateral and contralateral beta (de)synchronization during unimanual force production. Eur. J. Neurosci. 36, 2088–2097 10.1111/j.1460-9568.2012.08122.x 22583034

[B223] van Wijk B. C. M. Daffertshofer A. Praamstra P. (2008). Local and long-range beta synchrony in motor control, in Biomagnetism: Interdisciplinary Research and Exploration, eds Kakigi R. Yokosawa K. Kuriki S. (Sapporo/Japan: Hokkaido University Press), 1642–1648

[B224] van Wijk B. C. M. Daffertshofer A. Roach N. Praamstra P. (2009). A role of beta oscillatory synchrony in biasing response competition? Cereb. Cortex 19, 1294–1302 10.1093/cercor/bhn174 18836098

[B225] Vardy A. N. van Wegen E. E. H. Kwakkel G. Berendse H. W. Beek P. J. Daffertshofer A. (2011). Slowing of M1 activity in Parkinson's disease during rest and movement—an MEG study. Clin. Neurophysiol. 122, 789–795 10.1016/j.clinph.2010.10.034 21109487

[B226] Varela F. Lachaux J. P. Rodriguez E. Martinerie J. (2001). The brainweb: phase synchronization and large-scale integration. Nat. Rev. Neurosci. 2, 229–239 10.1038/35067550 11283746

[B227] Wang H. C. Lees A. J. Brown P. (1999). Impairment of EEG desynchronisation before and during movement and its relation to bradykinesia in Parkinson's disease. J. Neurol. Neurosurg. Psychiatry 66, 442–446 10.1136/jnnp.66.4.442 10201414 PMC1736289

[B228] Weinberger M. Mahant N. Hutchison W. D. Lozano A. M. Moro E. Hodaie M. Lang A. E. Dostrovsky J. O. (2006). Beta oscillatory activity in the subthalamic nucleus and its relation to dopaminergic response in Parkinson's disease. J. Neurophysiol. 96, 3248–3256 10.1152/jn.00697.2006 17005611

[B229] Whittington M. A. Traub R. D. Jefferys J. G. R. (1995). Synchronized oscillations in interneuron networks driven by metabotropic glutamate-receptor activation. Nature 373, 612–615 10.1038/373612a0 7854418

[B230] Wickens J. Hyland B. Anson G. (1994). Cortical cell assemblies—a possible mechanism for motor programs. J. Mot. Behav. 26, 66–82 10.1080/00222895.1994.9941663 15753061

[B231] Williams E. R. Baker S. N. (2009). Circuits generating corticomuscular coherence investigated using a biophysically based computational model. I. Descending systems. J. Neurophysiol. 101, 31–41 10.1152/jn.90362.2008 19019981 PMC2637020

[B232] Williams E. R. Soteropoulos D. S. Baker S. N. (2009). Coherence between motor cortical activity and peripheral discontinuities during slow finger movements. J. Neurophysiol. 102, 1296–1309 10.1152/jn.90996.2008 19474171 PMC2724360

[B233] Williams E. R. Soteropoulos D. S. Baker S. N. (2010). Spinal interneuron circuits reduce approximately 10-Hz movement discontinuities by phase cancellation. Proc. Natl. Acad. Sci. U.S.A. 107, 11098–11103 10.1073/pnas.0913373107 20534484 PMC2890710

[B234] Wingeier B. Tcheng T. Koop M. M. Hill B. C. Heit G. Bronte-Stewart H. M. (2006). Intra-operative STN DBS attenuates the prominent beta rhythm in the STN in Parkinson's disease. Exp. Neurol. 197, 244–251 10.1016/j.expneurol.2005.09.016 16289053

[B235] Witham C. L. Riddle C. N. Baker M. R. Baker S. N. (2011). Contributions of descending and ascending pathways to corticomuscular coherence in humans. J. Physiol. (Lond.) 589, 3789–3800 10.1113/jphysiol.2011.211045 21624970 PMC3171886

[B236] Witte M. Patino L. Andrykiewicz A. Hepp-Reymond M. C. Kristeva R. (2007). Modulation of human corticomuscular beta-range coherence with low-level static forces. Eur. J. Neurosci. 26, 3564–3570 10.1111/j.1460-9568.2007.05942.x 18052988

[B237] Womelsdorf T. Schoffelen J. M. Oostenveld R. Singer W. Desimone R. Engel A. K. Fries P. (2007). Modulation of neuronal interactions through neuronal synchronization. Science 316, 1609–1612 10.1126/science.1139597 17569862

[B238] Zhang Y. Chen Y. Bressler S. L. Ding M. (2008). Response preparation and inhibition: the role of the cortical sensorimotor beta rhythm. Neuroscience 156, 238–246 10.1016/j.neuroscience.2008.06.061 18674598 PMC2684699

